# Incorporating a Screening-Level Risk Quotient (RQ_screen) for Assessing Human Health Risk of Pharmaceutical Residues in Consumption Water

**DOI:** 10.3390/ijerph23070838

**Published:** 2026-06-25

**Authors:** Gabriel Souza-Silva, Igor F. C. Santos, Inês B. Gomes, Manuel Simões, Micheline R. Silveira, Vítor J. P. Vilar, Ana I. Gomes

**Affiliations:** 1 Faculty of Pharmacy, Federal University of Minas Gerais, Belo Horizonte 31270-901, Brazil; gabrielsouzasilva@ufmg.br (G.S.-S.);; 2 Sergio Arouca National School of Public Health, Oswaldo Cruz Foundation, Rio de Janeiro 21041-210, Brazil; 3 Laboratory for Process Engineering, Environment, Biotechnology and Energy, ALiCE, Faculty of Engineering, University of Porto, 4200-465 Porto, Portugal; ibgomes@fe.up.pt (I.B.G.);; 4 Laboratory of Separation and Reaction Engineering-Laboratory of Catalysis and Materials, ALiCE, Faculty of Engineering, University of Porto, 4200-465 Porto, Portugal

**Keywords:** aquatic environments, contaminants of emerging concern, human health risk assessment, One Health, pharmaceutical residues

## Abstract

**Highlights:**

**Public health relevance—How does this work relate to a public health issue?**

- Pharmaceutical residues are widely detected in drinking and environmental waters, representing an emerging pathway of chronic human exposure.
- The study addresses human health risks associated with multi-compound, low-dose exposure through water consumption within a One Health framework.

**Public health significance—Why is this work of significance to public health?**

- It integrates occurrence data across bottled, tap, and surface waters with risk assessment approaches (RQ and RQ_screen), enabling a comprehensive evaluation of exposure scenarios.
- It identifies vulnerable populations, particularly infants and children, as having higher relative exposure risks due to physiological and consumption factors.

**Public health implications—What are the key implications or messages for practitioners, policy makers and/or researchers in public health?**

- Screening-level prioritization (RQ_screen) highlights pharmaceuticals that require continuous monitoring even when traditional risk metrics indicate low concern.
- The findings support the need for risk-based water quality management, age-specific assessments, and policies targeting pharmaceutical contamination under the One Health approach.

**Abstract:**

Pharmaceutical residues are increasingly detected in aquatic environments and are recognized as contaminants of emerging concern. This systematic literature review compiled and evaluated published concentrations of pharmaceutical residues in bottled water, tap water, and surface water in Portugal, applying risk quotient (RQ) and screening-level risk quotient (RQ_screen) approaches to evaluate potential human health risks and prioritize contaminants. Assessment based on the compiled literature data across age groups showed bottled and tap water posed low risk, while surface water presented the highest concern, with compounds spanning the full risk spectrum. Key contributors to potential human health risk included hormones (17-alpha-ethinylestradiol, 17-beta-estradiol, estrone), ramipril, betamethasone, citalopram, and amoxicillin. RQ_screen highlighted compounds relevant for ongoing monitoring even in treated waters, such as carbamazepine, diclofenac, salicylic acid, warfarin, fluoxetine, and erythromycin, due to their persistence and toxicological significance. Both RQ and RQ_screen indicated higher risk values for infants and children, reflecting lower body weight and higher water intake per unit mass, underscoring the need for age-specific evaluations. The RQ_screen method proved useful for contaminant prioritization, identifying substances relevant for monitoring despite low concentrations. Overall, this systematic review highlights pharmaceutical residues as an emerging public and environmental health concern in Portugal and emphasizes the importance of targeted monitoring and risk-based management within a One Health framework.

## 1. Introduction

The global increase in pharmaceutical consumption [1], driven by population ageing [2], expanded access to pharmacological therapies, and the extensive use of pharmaceuticals in veterinary medicine and animal production systems [3], has contributed to the continuous release of pharmaceutical residues, defined as active pharmaceutical ingredients, their metabolites, transformation products, and other drug-related compounds originating from human and veterinary use, into the environment [4]. Unlike classical chemical contaminants, these compounds are intentionally designed to exert biological activity and, once introduced into ecosystems, may persist and interact across multiple environmental compartments [5]. Pharmaceutical residues are acknowledged as contaminants of emerging concern due to their pervasive occurrence in aquatic systems and their possible effects on environmental and public health [6].

Aquatic systems play a central role in the dissemination of these contaminants [7]. Following human and animal consumption, a significant fraction of pharmaceuticals is excreted either unchanged or as biologically active metabolites [8], ultimately reaching wastewater treatment plants that are often not designed to ensure their complete removal [9]. Accordingly, pharmaceutical residues have been commonly observed in surface water bodies, which are often used as sources for public water supply, as well as in tap water and bottled water, indicating that conventional treatment processes do not constitute an absolute barrier to their presence in water intended for human consumption [10].

Although reported concentrations are generally in the ng/L to µg/L range, human exposure occurs in a chronic, involuntary, and multicomponent manner, as multiple pharmaceuticals can coexist simultaneously within the same aquatic matrix [11]. This continuous exposure raises public health concerns, particularly for vulnerable populations such as children, the elderly, and individuals with preexisting health conditions [12]. Furthermore, the persistent presence of antibiotics in aquatic environments has been linked to the promotion of antimicrobial resistance [13], while hormones and other compounds with endocrine activity [14] raise concerns regarding long-term subclinical effects.

The issue of pharmaceutical residues in water is framed within the One Health paradigm [15], which recognizes the interdependence of human, animal, and environmental health. The use of pharmaceuticals across clinical, veterinary, and agricultural contexts, combined with improper disposal and the persistence of these compounds throughout the hydrological cycle, establishes a direct link between human practices, environmental degradation, and health risks [16]. In this context, water serves as a critical integrative vector, functioning simultaneously as a transport medium, environmental reservoir, and exposure pathway for humans and other organisms [17].

Despite the growing number of studies on the occurrence of pharmaceutical residues in aquatic environments, integrated assessments comparing different water matrices from a human exposure perspective remain limited [18]. Most research has focused on surface waters or effluents, with less attention given to the presence of these compounds in water consumed by the population and to systematic comparisons between tap and bottled water [19]. Furthermore, few studies incorporate human health risk assessments across life stages, a critical aspect for more realistic population-level analyses [20].

From an environmental health and sustainability perspective, this issue can also be understood through the One Health approach, which recognizes the interconnectedness of human, animal, and environmental health and promotes collaborative efforts to achieve optimal health outcomes across these domains. The issue aligns with the principles established by the 2030 Agenda for Sustainable Development Goal, particularly SDG 3 (Good Health and Well Being), SDG 6 (Clean Water and Sanitation), and SDG 12 (Responsible Consumption and Production), which emphasize the need to strengthen environmental management, enhance monitoring of micropollutants, and promote responsible practices throughout the pharmaceutical life cycle [21]. In this context, understanding the distribution levels and associated risks of these residues in Portuguese aquatic environments is essential to support regulatory frameworks, guide public policy, and inform mitigation strategies.

By combining environmental occurrence data with risk assessment metrics and the prioritization of pharmaceutical contaminants within a One Health framework, this study a thorough evaluation of pharmaceutical residues in bottled, tap, and surface waters in Portugal and employs a screening-level risk quotient (RQ_screen), a dimensionless index that ranks compounds by the ratio of their measured environmental concentration to a simplified reference value, intended for comparative prioritization rather than absolute risk estimation. By integrating occurrence data across multiple water matrices and accounting for differences among age groups and exposure scenarios, this study pinpoints priority pharmaceuticals that require further investigation and targeted management within water treatment and monitoring programs, thereby supporting water quality management and public health protection, in line with the objectives of the 2030 Agenda.

## 2. Materials and Methods

### 2.1. Selection of Studies

**Figure 1:**
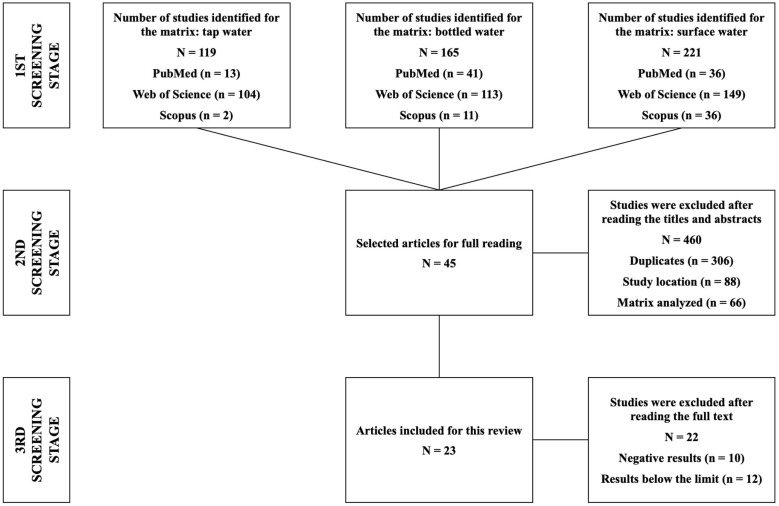
Flowchart of the literature search, screening, eligibility assessment, and study selection process used to identify studies reporting pharmaceutical residues in Portuguese waters. Flow diagram illustrating the systematic literature search and study selection process adopted in this review. Records were identified through searches in PubMed, Web of Science, and Scopus for three environmental matrices relevant to human exposure to pharmaceutical residues: tap water, bottled water, and surface water.

A systematic search for studies reporting the occurrence of pharmaceutical residues in Portuguese waters was conducted in the PubMed, Web of Science, and Scopus databases (Figure 1). The following search terms were used combined with Boolean operators: “tap water” AND pharmaceuticals AND Portugal; (“drinking water” OR “bottled water”) AND pharmaceuticals AND Portugal; and “surface water” AND pharmaceuticals AND Portugal.

The study selection followed three steps: (1) screening of titles and abstracts, (2) full-text review, and (3) data extraction of reported concentrations for each water matrix. Articles published since 2010 and in English or Portuguese were included, provided they reported quantitative data on pharmaceutical detection in Portuguese waters. Only studies with clearly specified detection limits and validated analytical methodologies were considered eligible. Studies reporting extrapolated data and/or environmental estimates of pharmaceutical compound concentrations in water were excluded from this review.

### 2.2. Data Collection

Following the selection of eligible studies, pharmaceutical residue concentration data were extracted separately for each water matrix (Table S1). For bottled water, studies analyzing commercially bottled water in Portugal were included. Reported concentrations were fully extracted to represent the actual level of exposure associated with the consumption of treated water intended for human use. For tap water, the minimum, mean, and maximum concentrations of each detected pharmaceutical in Portugal were compiled.

These data enabled the assessment of potential exposure scenarios from direct consumption of distributed water. Finally, for surface waters, studies analyzing rivers, reservoirs, and estuaries in Portugal were considered. For risk assessment purposes, direct consumption without treatment was assumed to represent an extreme exposure scenario. For each identified pharmaceutical, the extracted data were used in the risk assessment described in Section 2.3, including the calculation of risk quotients and DWEL values.

### 2.3. Human Health Risk Assessment (RQ)

The human health risk assessment was conducted through the calculation of risk quotients (RQs), considering different life stages to improve exposure characterization across 10-year age intervals. These age groups were defined based on the guidelines of the United States Environmental Protection Agency (EPA), as outlined in the document Guidance on Selecting Age Groups for Monitoring and Assessing Childhood Exposures to Environmental Contaminants. The adopted categories are detailed in Table 1 [22].

The RQs for the pharmaceutical residues analyzed in this review were calculated using the minimum, mean, and maximum concentrations, whenever available, measured in bottled, tap, or surface water samples, according to Equation (1). For all evaluated matrices, the lowest detected concentrations of the identified pharmaceuticals were considered the best-case scenario, the mean concentrations represented the near-realistic scenario, and the highest detected concentrations were classified as the worst-case scenario. When the RQ is less than 0.1, the risk is classified as low; values between 0.1 and 1.0 are considered moderate; and values greater than 1.0 are classified as high, suggesting a potential health risk associated with involuntary exposure through water ingestion [23].

(1) $$RQ=\frac{Cs}{DWEL},$$

In this equation, Cs corresponds to the minimum, mean, or maximum concentration of the pharmaceutical residue detected in the water sample (bottled, tap, or surface water), while the DWEL (Drinking Water Equivalent Level) values, expressed in μg/L, were determined using Equation (2), following the criteria described in the document Wyoming Water Rules and Regulations [24].

(2) $$DWEL=\frac{ADI\times BW\times HQ}{DWI\times AB\times FOE},$$

In this equation, ADI represents the Acceptable Daily Intake (µg/kg/day); BW refers to the 50th percentile body weight for each considered age group (kg); HQ is the Hazard Quotient, set to 1 to represent the threshold of acceptable risk, allowing the estimation of a maximum concentration in drinking water that would not pose a risk to human health; and DWI indicates the daily drinking water intake (L/day), with age-specific values obtained from the EPA Exposure Factors Handbook [22].

The gastrointestinal absorption rate (AB) was assumed to be 1, while FOE corresponds to the frequency of exposure (350 days/365 days = 0.96) [25]. Body weight and daily intake values are presented in Table 1. The ADI values represent the level of a given substance that is not expected to result in any adverse effect in a potentially exposed population, including susceptible subpopulations [26]. In this study, ADI values were obtained using Equation (3).

(3) $$ADI=\frac{RD}{UF},$$

Here, RD represents the reference dose corresponding to the lowest therapeutic effect reported in the drug label, and UF is the uncertainty factor, set to 1000, which results from the following: a factor of 10 to account for human response variability (intraspecies variation); a factor of 10 for the protection of sensitive subgroups, such as children and infants; and a factor of 10 to account for the fact that the reference dose for the lowest therapeutic effect is not a no-effect level [27]. When ADI values were not available in the literature for metabolites, i.e., pharmaceutical byproducts, the same ADI as the parent compound was applied. For pharmaceuticals with available literature data, these values were used as the starting point for the assessment (Table S2).

For compounds for which no ADI value was available in the literature [26,27,28,29,30,31,32,33,34,35,36,37,38,39,40,41,42,43,44,45] and for which no parent compound could be identified (e.g., illicit drugs such as cocaine and its metabolite benzoylecgonine), quantitative RQ-based assessment was not performed, as no established safe intake reference exists for these substances in drinking water matrices. These compounds were classified as “uncategorized” and are discussed qualitatively in Section 3.3. The absence of regulatory toxicological benchmarks for illicit drugs underscores a methodological limitation of RQ-based frameworks and reinforces the need for complementary screening approaches such as RQ_screen. A sensitivity analysis was conducted to evaluate the influence of compounds with high or uncertain ADI values on the overall risk ranking: excluding the top 10% of ADI values did not alter the classification of the high-risk compounds identified, indicating that the prioritization results are robust to uncertainty in individual ADI estimates.

### 2.4. Identification of Contaminants of Emerging Concern Through Prioritization Quotient

To identify contaminants of emerging concern for human health via water consumption and establish a ranking based on relative hazard, a prioritization index was developed by the authors specifically for the purposes of this literature review using a screening risk quotient (RQ_screen). This metric was intended exclusively for comparative screening, allowing compounds to be ranked according to the combination of their environmental occurrence and toxicological potency. The proposed RQ_screen approach represents a methodological tool created in this review to support the prioritization of contaminants based on available occurrence and toxicity data. Unlike conventional human health risk assessment approaches, RQ_screen does not provide an estimate of absolute health risk; instead, it was used to compare the relative relevance of detected compounds and to identify those requiring priority attention in monitoring programs, refined risk assessments, or management actions (Equation (4)).

(4) $$RQ\text{\_}screen=\frac{Cs}{WEL},$$

The WEL was calculated following the same structure as the DWEL (Equation (2)), but omitting the uncertainty factor (UF) incorporated in the ADI derivation. Specifically, WEL was determined using Equation (5) where RD is the reference dose corresponding to the lowest therapeutic effect reported in the drug label (µg/kg/day), and all other parameters retain the same definitions as in Equation (2). The omission of the UF (set to 1000 in the formal risk assessment) is justified by the purely comparative nature of RQ_screen: since the UF is a fixed multiplicative constant applied uniformly to all compounds, its inclusion or exclusion does not alter the relative ranking among substances. Its removal avoids the over-conservative distortion that would otherwise compress RQ_screen values across compounds and impair the discriminatory power of the prioritization. Accordingly, WEL values were derived directly from the RD values listed in Table S2, using the age-specific body weight and water intake parameters in Table 1.

(5) $$WEL=\frac{RD\times BW\times HQ}{DWI\times AB\times FOE}$$

In this equation, the RQ_screen is calculated as the ratio between the pharmaceutical compound concentration (Cs) in water, whether minimum, mean, or maximum, and a Water Equivalent Level (WEL). In contrast to DWEL (Equation (2)) which was used in the formal human health risk assessment described in Section 2.3, WEL was adopted here as a simplified reference value for comparative screening purposes. This procedure preserves the relative differences among compounds and avoids the over conservative distortion that may arise when multiple uncertainty assumptions are incorporated into a metric intended only for prioritization. This approach allows the direct use of analytically reported concentrations, preserving comparability between compounds for screening purposes.

RQ_screen values were used exclusively as a screening and prioritization tool. According to the adopted criteria, compounds with RQ_screen < 0.01 were classified as low-priority, values between 0.01 and 0.1 as moderate priority, values between 0.1 and 1.0 as high-priority, and RQ_screen ≥ 1.0 as critical-priority contaminants. These categories reflect relative prioritization based on the combined effect of environmental concentrations and toxicological margin. This classification was applied consistently across compounds and scenarios, allowing for an objective ranking of substances of greatest relevance for screening and prioritization purposes

## 3. Results and Discussion

### 3.1. Occurrence of Pharmaceutical Residues Across Water Matrices

#### 3.1.1. Bottled Water

Bottled water showed the lowest concentrations among all matrices analyzed (Table 2). Based on the compiled data, the pharmaceutical residues detected in bottled water in Portugal (*n* = 9, pharmaceutical residues) with the highest average concentrations were salicylic acid (25.9 ng/L), carbamazepine (12.7 ng/L), and warfarin (7.6 ng/L). Among these, salicylic acid deserves particular attention, not only because of its concentration in this matrix, but also because it is both an active ingredient in topical formulations and a major metabolite and transformation product of acetylsalicylic acid, one of the most widely consumed analgesic and anti-inflammatory drugs worldwide [46]. Carbamazepine and warfarin, in turn, are an antiepileptic and an anticoagulant under continuous use, respectively [47,48].

Diclofenac was also recurrently detected in bottled water, although at lower average concentration than salicylic acid, carbamazepine, and warfarin. Its occurrence is noteworthy because it is one of the most widely consumed and readily available drugs for human use [70]. However, the compiled data show that consumption alone is insufficient to explain the occurrence of pharmaceutical residues in this matrix. Physicochemical behavior and environmental stability also contribute to the final concentration profile [71]. This is illustrated by the contrast between diclofenac and salicylic acid.

Diclofenac is more susceptible to photodegradation, with a reported half-life (*t*_1/2_) of up to 2 h [72], whereas salicylic acid is more stable, with a *t*_1/2_ extending up to 168 h [73]. Thus, despite its broader consumption and commercialization, diclofenac is more rapidly degraded in the environment, whereas salicylic acid has greater stability and, consequently, higher environmental concentration. These findings confirm the persistence of pharmaceutical residues along the water supply chain and indicate that even treated waters are not free from contamination, reinforcing the need to include this matrix in integrated human exposure assessments [74].

#### 3.1.2. Tap Water

In addition to bottled water, tap water is consumed by the Portuguese population [75], highlighting the relevance of analytical studies addressing pharmaceutical residues in this matrix (Table 2). The compiled data for tap water revealed eight detected pharmaceutical compounds. A particularly relevant finding was the presence of illicit substances in treated water distributed to the population, a pattern also reported in other European countries, including Spain, France, Belgium, Germany, Italy, Turkey, and Luxembourg [51]. Cocaine and its active metabolite benzoylecgonine were detected at the highest average concentrations in Portuguese tap water [51], followed by salicylic acid, carbamazepine, and diclofenac. This distinguishes tap water from bottled water not only in concentration profile, but also in the nature of the detected compounds.

Apart from the illicit substances, the ranking of the most concentrated pharmaceutical residues in tap water broadly resembled that observed in bottled water, particularly for salicylic acid, carbamazepine, diclofenac, and warfarin. Even so, the relative order of these compounds differed between the two matrices. Diclofenac, presented higher average concentration in tap water than in bottled water, whereas warfarin showed the opposite trend. These differences are likely related to matrix-specific source and transport conditions, including variations in contamination sources (e.g., wastewater treatment plant effluents, agricultural runoff, and industrial discharges), hydrological characteristics, dilution capacity, residence time, and physicochemical processes affecting contaminant mobility and persistence in each aquatic matrix, rather than to a single common pathway.

Diclofenac, with higher lipophilicity (logKow = 4.51), may be more affected by soil retention processes than warfarin (logKow = 2.70), which could contribute to lower transfer to bottled waters derived from groundwater sources, as reported in the studies included in this review. Therefore, the soil may act as a natural filter, decreasing the amount of diclofenac reaching deep aquifers [76]. By contrast, the higher occurrence of warfarin in bottled water is consistent with its broader use as both an anti-inflammatory and a rodenticide in agricultural, industrial, and urban areas, which may favor diffuse environmental inputs beyond the wastewater pathway [77,78]. These interpretations should nevertheless be viewed cautiously, since the comparison is based on compiled data from different studies rather than on paired source-to-distribution datasets.

Salicylic acid and carbamazepine also presented higher concentrations in tap water compared to bottled water, with an approximate 2.1-fold increase for salicylic acid (52.7 ng/L vs. 25.9 ng/L) and a 1.2-fold increase for carbamazepine (15.2 ng/L vs. 12.7 ng/L). This pattern, together with the recurrent detection of carbamazepine, diclofenac, salicylic acid, and warfarin in tap water across different tap water studies shows that conventional treatment does not ensure their complete removal [79]. In this respect, tap water represents a distinct and relevant matrix for exposure assessment, as it integrates both source-water contamination and the incomplete elimination of selected pharmaceutical residues during treatment and distribution.

#### 3.1.3. Surface Water

Among the evaluated matrices, surface water showed the highest number of studies (*n* = 19) and analytical determinations (*n* = 919) comprising various pharmaceutical residues (*n* = 76) and distinct therapeutic groups (*n* = 31) (Table 2). This matrix receives a large portion of pharmaceutical residues used by humans, whether from urban, hospital, agricultural, or industrial sources [80,81]. The compounds, with the highest average concentrations were amoxicillin (6416.7 ng/L), omeprazole (3223.0 ng/L), and tramadol (1625.6 ng/L). Amoxicillin, although a controlled-use drug, is the most widely consumed antibiotic globally, either alone or in combination with other antibiotics [82]. Its high occurrence is consistent with the combined influence of consumption patterns, water solubility (logKow= 0.87, water solubility = 3000 mg/L), and reported persistence (*t*_1/2_ = 28 days) [83]. Omeprazole and tramadol likewise stood out because of their elevated concentrations relative to most other compounds detected in this matrix.

It should be noted that the concentration dataset compiled for surface water spans studies conducted over different periods, using distinct analytical methods and sampling strategies. Some recent studies, particularly those employing high-sensitivity mass spectrometry techniques, reported substantially higher concentrations for selected compounds (e.g., amoxicillin up to 15,382 ng/L [53]; omeprazole up to 8255 ng/L [53]; acetaminophen up to 10,587 ng/L [52,53]) compared to earlier investigations. These high-concentration values likely reflect improvements in analytical sensitivity and/or sampling near point-sources (e.g., hospital effluents, WWTP discharges) rather than a generalized increase in environmental contamination. Accordingly, worst-case scenarios reported here should be interpreted as representing localized or episodic peak exposures rather than representative ambient concentrations. The heterogeneity in study design across the compiled dataset is an inherent limitation of this type of systematic literature review and was addressed by presenting minimum, mean, and maximum concentration scenarios, thereby bracketing the likely range of exposure conditions.

The surface-water profile also shows that concentration cannot be interpreted only as a function of use. Tramadol, for example, was detected at much higher (10-times) average concentration than diclofenac, despite the broader consumption of diclofenac [84]. This difference is consistent with the combined effect of environmental behavior and treatment-related removal. Diclofenac is susceptible to photodegradation and has been reported to be more effectively removed in WWTPs (approx. 97%) than tramadol (c.a. 27%) [79,85,86]. The contrast between these two analgesics therefore illustrates that the occurrence profile in surface water reflects not only use, but also compound-specific persistence and removal behavior.

Beyond the highest concentration values, surface water also contained compounds with particularly frequent detection across studies, including carbamazepine (52.7 ng/L, *n* = 92), paracetamol (256.11 ng/L, *n* = 77), fluoxetine (5.7 ng/L, *n* = 69), and salicylic acid (109.7 ng/L, *n* = 67), which had the highest numbers of analytical determinations. Although not all are included in the European Commission watch list [87], these residues represent different therapeutic classes, consumption patterns, epidemiological relevance, behavior in WWTPs, and environmental marker potential [88]. According to European Commission 439/2025 [87], there is strong encouragement to monitor pharmaceutical residues with high persistence (e.g., carbamazepine), high population consumption (e.g., paracetamol and salicylic acid), and high ecotoxicological risk (e.g., fluoxetine). In this respect, surface water emerges as the matrix that best captures the breadth and continuity of pharmaceutical contamination in the Portuguese aquatic environment.

### 3.2. Integrated Comparison Across Water Matrices

Among the data collected in this study, only four pharmaceutical residues—carbamazepine, diclofenac, salicylic acid, and warfarin—were present in all evaluated matrices, while another four—sulfadiazine, sulfapyridine, erythromycin, and atenolol—were found in two matrices (bottled and surface water). This limited overlap constrained the integrated comparison to a small set of compounds and required cautious interpretation, particularly because the reported concentrations were compiled from different studies with distinct analytical methods, sampling strategies, and environmental conditions.

To support a more consistent cross-matrix comparison, an Across-Matrix Factor (AF) was considered, which expresses the concentration ratios between surface water and tap water matrices. The AF was used exclusively as a comparative metric to describe relative concentration differences between matrices and should not be interpreted as a measure of contaminant attenuation, removal efficiency, or treatment performance. In general, the obtained values showed that concentrations tended to be higher in surface water than in tap and bottled water, although the magnitude of these differences varied markedly among compounds (Table 3). Within this context, the comparison was used to identify broad distribution patterns across matrices, rather than to infer treatment efficiencies or direct attenuation pathways.

The strongest cross-matrix contrasts were associated with compounds whose physicochemical properties favor environmental attenuation or greater susceptibility to reduction before reaching consumption waters. Diclofenac and sulfadiazine fall within this group, which is consistent with their sensitivity to photodegradation (*t*_1/2_ = 2.3 h and 1.7 h, respectively) [72,89].

In the case of diclofenac, adsorption onto organic matter may also contribute to the lower concentrations observed outside surface waters [90]. Atenolol followed the same overall pattern, although likely for different reasons. Its high-water solubility (26,700 mg/L), low logKow (0.16), and predominantly cationic character at environmental pH favor interactions with suspended matter and flocs, which may facilitate reduction during conventional coagulation and filtration processes used in water and wastewater treatment. Within the limits of this cross-study comparison, these compounds are therefore associated with the most pronounced concentration decreases across matrices.

By contrast, carbamazepine, salicylic acid, and erythromycin showed more limited cross-matrix differentiation, pointing to greater persistence throughout the aquatic cycle. This is particularly consistent with carbamazepine, whose recurrent detection across environmental and drinking water matrices is well established.

Salicylic acid and erythromycin also tend to be less affected by photodegradation and conventional treatment processes. In the case of erythromycin, its complex macrolide structure favors the formation of equally stable transformation products, hindering complete removal [91]. The comparatively smaller concentration contrasts observed for these compounds support their continued transfer across water matrices, including waters intended for human consumption.

Warfarin differed from all other compounds by showing a distinct occurrence profile, with no clear decrease toward bottled water. This pattern does not support interpretation in terms of sequential attenuation across matrices. Considering that most bottled waters originate from groundwater, it is plausible that leaching and percolation processes have a greater influence, favoring the presence of this compound in aquifers. Its distribution is therefore better interpreted in light of source-related differences than as a treatment-related effect, which is also coherent with the broader discussion in Section 3.1 regarding its possible association with diffuse environmental inputs.

### 3.3. Human Health Implications of Pharmaceutical Residues

Considering the concentrations of pharmaceutical residues detected in bottled and tap water, all compounds presented RQ values below 0.1, even when the highest detected concentrations were considered, indicating low risk according to the adopted classification criteria. The highest RQ values identified were for carbamazepine and warfarin, with RQ = 0.010 for both. This low-risk profile was maintained across all age groups, meaning that even children (<10 years) and the elderly (>85 years) had RQ values below 0.1.

On the other hand, untreated surface water was associated with higher RQ values due to the presence of pharmaceutical residues at elevated concentrations. Compounds with the highest RQ values were ramipril (RQ = 185.9), 17-alpha-ethinylestradiol (RQ = 25.7), betamethasone (RQ = 11.0), citalopram (RQ = 10.7), lorazepam (RQ = 8.7), furosemide (RQ = 7.4), omeprazole (RQ = 6.1), 17-beta-estradiol (RQ = 5.1), estrone (RQ = 4.4), and amoxicillin (RQ = 3.9). Table 4 presents the pharmaceutical compounds classified according to their potential risk to human health across all age groups evaluated in this study.

Among the compounds classified as high risk, steroid hormones predominate (17-alpha-ethinylestradiol, 17-beta-estradiol, estrone) and are characterized by very low ADI values (µg/kg/day) (<0.001). Therefore, even low concentrations in water (<20.0 ng/L) are sufficient to result in RQ values above 1.0. Other studies have likewise reported that hormones in drinking water may pose potential risks to human health even at concentrations in the ng/L range [14]. Although direct effects on humans remain insufficiently quantified, their environmental persistence and chronic exposure make these compounds particularly relevant in health risk assessment [14,92].

Beyond hormonal compounds, the antihypertensive ramipril stands out as a pharmaceutical residue associated with the highest RQ in Portuguese surface waters. In addition to its low ADI value (0.0018 µg/kg/day), this compound was reported at high environmental concentrations (2659.00 ng/L), which explains its position in the ranking. In a study conducted in Canada [28], evaluating 335 potentially waterborne pharmaceuticals, ramipril and its metabolite ramiprilat were classified among the compounds of greater concern for human health. Even so, the lack of consistent detections in drinking water raises uncertainty when extrapolating this result beyond the surface-water scenario considered here.

Not all compounds detected at high concentrations in surface water were associated with high RQ values. Tramadol, ibuprofen, and naproxen were detected at high concentrations in surface water (Table 2), yet all presented RQ values below 0.1. This shows that the final RQ is strongly conditioned by the toxicological reference value and not by concentration alone. Consistent with these observations, a study conducted in water supply systems in Lisbon (Portugal) evaluated 31 pharmaceuticals in raw and treated water, with concentrations ranging from 0.005 to 46 ng/L [25]. Even for the compounds with the highest detected concentrations, the RQ-based assessment yielded extremely low values (<0.001). Together, these findings show that the presence of pharmaceutical residues at measurable levels in drinking water does not necessarily represent a relevant risk to human health, and that the toxicological properties of each compound strongly condition the final RQ.

Although RQ-based analysis allows the identification of compounds with greater potential concern in surface waters, the interpretation of these results requires caution [29]. The ranking is highly dependent on the toxicological benchmark adopted for each compound, which complicates direct comparison across therapeutic classes. In this context, low ADI values are particularly relevant for steroid hormones, antidepressants, and benzodiazepines, whereas common analgesics, antibiotics, and NSAIDs tend to present intermediate ADIs, and radiological contrast agents much higher values [25]. Accordingly, RQ should be interpreted primarily as a screening tool for relative concern under the adopted assumptions, rather than as a direct measure of actual exposure burden.

Considering only the RQ values, the results indicate low immediate risk for human exposure to pharmaceutical residues through bottled and tap water in Portugal. However, some compounds, such as cocaine and its metabolites, could not be assessed through this approach. Beyond this limitation, RQ has another constraint as a sole evaluation parameter: it does not account for cumulative effects, interactions between different pharmaceuticals, chronic exposure, or environmental persistence. Furthermore, the interpretation is limited to the matrices evaluated, without considering potential contamination peaks or impacts on aquatic ecosystems [29].

The detection of cocaine and its metabolites in tap water represents a particular challenge for risk assessment, as no safe consumption limits have been established for these substances in drinking water matrices. This exposes a clear limitation of RQ-based approaches, which rely on predefined toxicological reference parameters. Additionally, the presence of these compounds, even at low concentrations, raises concerns regarding chronic exposure, cumulative effects, and potential impacts on vulnerable populations, reinforcing the need for continuous monitoring and complementary screening approaches, such as RQ_screen, to support the prioritization of contaminants of concern without clear regulatory or toxicological benchmarks.

### 3.4. Prioritization of Contaminants of Concern (RQ_screen)

Pharmaceuticals analyzed in this study presented widely varying toxicological sensitivity, reflected in ADI values spanning several orders of magnitude (Table S2). Among the compounds identified and analyzed, only those present in surface water posed a potential risk to human health based on RQ. However, the absence of RQ-based concern in bottled and tap water does not preclude the need for prioritization, particularly when screening approaches are intended to identify compounds that remain relevant despite low measured concentrations.

As observed in the human health risk assessment, ADI values strongly influence the risk associated with pharmaceutical residues in water, together with their environmental persistence [93,94]. Therefore, RQ_screen was calculated without the uncertainty factor incorporated into ADI, namely the components related to variability in human response, protection of sensitive subgroups such as children and infants, and the uncertainty of the dose lacking a defined no-effect level. Under this framework, RQ_screen was used as a prioritization metric rather than as a formal risk estimate.

Considering the minimum, average, and maximum concentrations of pharmaceutical residues detected in bottled and tap water (Table 5 and Table 6), several compounds remained relevant for monitoring despite the low RQ values obtained for these matrices. In bottled water, carbamazepine, diclofenac, erythromycin, salicylic acid, and warfarin consistently emerged as high or critical priorities, reflecting the combination of occurrence, persistence, toxicological relevance, and the greater susceptibility of younger population groups.

In tap water, all compounds identified and evaluated in this matrix showed relevance in terms of monitoring, since for the newborn age group (<1 year), all presented moderate to high RQ_screen values (Table 6). Carbamazepine stands out, exceeding an RQ_screen of 1.0 across all age groups in both realistic and worst-case scenarios, thus classified within the critical priority category. Diclofenac and warfarin also showed elevated RQ_screen values in infants and children, while salicylic acid consistently remained a high-priority compound. These results show that decreases in concentrations do not directly translate into proportional reductions in prioritization, emphasizing the need for screening approaches that do not rely solely on concentration levels [74,95].

Surface water demonstrated the broadest prioritization spectrum, with compounds distributed across all RQ_screen categories: low priority (8 compounds), moderate (11 compounds), high (22 compounds), and critical priority (33 compounds, including carbamazepine, diclofenac, steroid hormones, antibiotics, and antidepressants) (Table 7). The broad spectrum illustrates the complexity of contamination profiles in this matrix and reinforces its role as a sentinel for early detection of high-risk contaminants [96,97]. In contrast with bottled and tap water, where prioritization is concentrated in a smaller group of recurrent compounds, surface water combines high occurrence, greater chemical diversity, and a wider spread of toxicological profiles. This makes it the matrix that most clearly captures the breadth of pharmaceutical contamination and the range of compounds requiring differentiated monitoring attention.

Comparative analysis across matrices revealed a restricted subset of compounds consistently classified as high-priority, independent of water type or exposure scenario. These compounds, including carbamazepine, diclofenac, salicylic acid, warfarin, fluoxetine, and erythromycin, are characterized by recurrent detection, environmental persistence, and compound-specific attenuation along the water cycle, indicating that monitoring only treated waters may overlook contaminants still relevant in surface waters [98,99]. Their recurrence across multiple matrices supports their classification as contaminants of critical concern and the need for targeted environmental management and monitoring strategies [100].

Given the persistence of these priority compounds, age-specific exposure assessment is critical. Stratified analyses revealed systematic gradients, with the highest RQ_screen values observed in infants (0–1 year) and children (1–10 years), followed by a gradual decline in older age groups [101,102].

This pattern reflects physiological differences, including lower body weight and higher water consumption per unit mass in younger populations, which amplify exposure relative to older individuals [103,104]. Even in adults and the elderly, however, compounds with low ADI values, such as hormones and antidepressants, remained relevant within the prioritization framework.

These findings highlight the need for proactive and risk-based monitoring strategies. Within this context, the integration of pharmaceutical products into routine water quality monitoring, the establishment of priority watch lists, and the adoption of risk-based prioritization approaches can support early detection of contaminants of high or critical concern and guide preventive management strategies, in line with the One Health framework [105,106,107,108,109]. For monitoring in Portugal, this implies an integrated prioritization strategy combining regulatory relevance, environmental persistence, and risk potential (Table 8).

Substances such as ciprofloxacin, fluconazole, and propranolol stand out due to their recent inclusion in the EU Watch List [110], reflecting current regulatory concerns. In parallel, compounds such as estradiol, estrone, and diclofenac, although not present in the most recent lists, remain highly relevant due to their well-documented ecotoxicological effects, particularly endocrine disruption and aquatic toxicity [111].

Additionally, carbamazepine remains a classic marker of environmental persistence, being frequently detected in different aquatic matrices and with limited removal during wastewater treatment [112]. Taken together, these criteria show that the selection of priority contaminants should not rely exclusively on regulatory listing, but also on the strength of the evidence supporting persistence, occurrence, and environmental impact.

In line with these considerations, comparing our prioritization with Directive (EU) 2024/3019 reinforces its relevance [113]. The directive mandates monitoring and >80% removal efficiency for at least six pharmaceuticals from a predefined list in wastewater treatment plants. Furthermore, a comparison with the treatment categories from Directive (EU) 2024/3019 [113] reveals notable overlaps with our RQ_screen prioritization.

Of the eight substances in Category 1 (easily treatable), six: carbamazepine, clarithromycin, diclofenac, hydrochlorothiazide, citalopram, and venlafaxine, are present in our study, with four classified as critical (RQ_screen > 1.0) and two as high (0.1–1.0). None from Category 2 appear in our dataset. This alignment underscores the high risk of several easily treatable compounds, suggesting that RQ_screen effectively identifies regulatory priorities while spotting potential gaps where treatability does not mitigate environmental concern.

### 3.5. Health and Ecological Implications of Priority Pharmaceutical Contaminants

The compounds found in the high-risk group, ciprofloxacin and fluconazole, present big worries beyond just being toxic. Anti-microbial resistance is now seen as a serious threat to people and animals alike, and the environment plays a key role in how anti-microbial resistance spreads. Ciprofloxacin is especially problematic because it can positively select for resistance integrins (intI1) at concentrations commonly found in our surroundings. This means that even mild levels of ciprofloxaxin in water sources can influence microorganisms negatively. Studies have shown that contact with less-than-inhibitory concentrations of ciprofloxacin can result in steady, low-level multi-drug resistance in *Escherichia coli*. The development of this resistance is influenced by both time and dose. Similarly, fluconazole, which is used against fungi, fuels worries about resistant Candida strains in those with weakened immune systems who rely on such drugs for basic protection [114,115,116].

The moderate-risk group, which includes estrone, estradiol, propranolol, diclofenac, and carbamazepine, contains compounds known for their non-target biological effects. Estrogenic activity from these compounds can boost risks of cardiovascular disease and breast and prostate cancer in people. It also leads to issues like feminization in male fish and messed-up reproductive traits in other animals. Estrogenic hormones, such as estrone and estradiol, are known endocrine disruptors. They can affect both aquatic life and our health at super tiny concentrations—think 0.007 ng/L in drinking water for both estrone and estradiol. That’s way too small for current treatment systems to handle all the time. According to the Endocrine Society, the scientific consensus is that these disrupting compounds are linked to lots of chronic diseases. These include troubles with neurodevelopment, reproduction, metabolism, and even some types of cancer [21,117].

Propranolol, a non-selective beta-blocker, poses risks to non-target creatures through its cardiovascular effects. Fish embryos showed significant bradycardia and deformed hearts when exposed to just 0.09 µg/L of propranolol. Zebrafish were hit hard too, making us think these issues might apply to other vertebrates. Diclofenac, an often-found non-steroidal anti-inflammatory, shows kidney and liver damage in wildlife at normal enviro concentrations. Mice chronically exposed to this stuff plus mixtures in drinking water faced delayed puberty in males and early puberty in females, and so did their kids. This highlights the big problem of intergenerational effects from low-level combos. Carbamazepine’s trouble starts during pregnancy. It raises the risk of restricted growth and birth defects if mom’s got therapeutic levels. Environmental levels are way lower, but the drug’s near-ubiquity and treatment-resistance in water mean we can’t let our guards down [118,119,120].

These findings show that relying only on direct toxicity levels to assess risks might actually be underestimating health issues linked to constantly drinking water with pharmaceutical residue. Especially for hormone-related substances, small changes in concentration can lead to big effects on how our bodies react to them, even when those doses are low. This is tricky because it makes setting a safe exposure limit tough. Plus, if people get exposed to endocrine disruptors at low doses, particularly when they’re developing in the womb or as infants, it could alter their growth via epigenetic and transgenerational routes. So, the approach used here does a crucial job in figuring out which stuff we urgently need to keep an eye on and handle in water treatment plants [121,122].

This framework’s importance is also backed by its alignment with results from various regions around the world. This shows that the high-risk compounds aren’t just local issues but have broader relevance, beyond any single geographic area. In European studies on rivers that cross country boundaries, carbamazepine, diclofenac, and 17α-ethinylestradiol often exceed safe levels for the environment. This matches our results too, since these same chemicals got high risk quotient scores in our study. In freshwater systems in central Europe, experts also flagged diclofenac, estrone, and estradiol as big worries due to their high risk quotients. These findings are pretty similar to our moderate-to-high risk classifications. Regulatory actions back up these concerns; certain chemicals like diclofenac, 17β-estradiol, and 17α-ethinylestradiol were early picks to be included on the EU Water Framework Directive Watch List [110]. Even more antibiotics, such as amoxicillin and ciprofloxacin, got added in 2018 because they’ve become widespread issues across the EU [123,124].

Across various parts of the world, similar findings keep popping up. In Brazil [27], for instance, a risk assessment for pharmaceuticals and endocrine-disrupting stuff in drinking water found seven risky compounds. Three were estrogens and four were anti-inflammatory drugs, matching what was seen in the current research. In China [23,125], studies showed that carbamazepine appeared in over 20% of tap water samples from 13 cities. They also noticed that antibiotics and antiparasitic drugs needed more focus, especially since they affect infants and kids heavily. These consistent international results show that the risky compounds found using the RQ_screen method aren’t unique to Portugal. Instead, they’re persistent and widespread issues across the globe. Because of this, there’s a stronger push for coordinated monitoring plans worldwide. It also highlights how useful the suggested prioritization approach really is.

## 4. Conclusions

This study demonstrates that pharmaceutical residues are associated with compound-dependent profiles across water matrices, with surface waters representing the most critical matrix within the evaluated conditions. In bottled and tap water, all compounds remained below the adopted RQ threshold for low risk, even under the highest detected concentrations. Yet the RQ_screen analysis showed that low concentrations in treated water do not necessarily place compounds outside monitoring priority, particularly when recurrent occurrence is combined with low toxicological thresholds. By integrating RQ and RQ_screen, this study established a framework that distinguishes compounds of immediate concern from those that, despite low measured concentrations, remain relevant for prioritization and surveillance.

Across matrices, some compounds remained consistently relevant due to the combination of persistence, toxicological significance, and recurrence. Carbamazepine and diclofenac were repeatedly retained within the priority group, while estradiol, estrone, ciprofloxacin, fluconazole, and propranolol also emerged as relevant targets for monitoring because they combine occurrence with endocrine activity, ecotoxicological relevance, persistence, or regulatory interest. These results show that concentration alone is not sufficient to define priority and that monitoring strategies should incorporate toxicological properties, environmental behavior, and age-dependent exposure patterns, particularly for infants and children, who systematically presented the highest RQ_screen values.

These findings must be interpreted within the limits of the study design. The analysis relied on concentrations reported in the literature and is therefore subject to spatial, temporal, and methodological variability among studies. Limited ADI data for some compounds, particularly metabolites and illicit drugs, prevented quantitative assessment within the same framework. The analysis was also restricted to ingestion through water and did not include other exposure routes, such as dermal contact, inhalation, or the consumption of aquatic organisms. Likewise, neither RQ or RQ_screen accounts for mixture effects or for temporal peaks in contamination, both of which may alter the actual exposure context.

Further studies should strengthen current assessment frameworks by using more consistent monitoring datasets, providing improved toxicological information on metabolites and transformation products, and including mixture effects and multiple exposure routes. Within this context, RQ_screen remains useful as a screening and prioritization tool, particularly under data-limited conditions, but its interpretation becomes more robust when supported by broader toxicological and exposure information. Taken together, the results support targeted, risk-based monitoring strategies and provide a structured basis for regulatory prioritization and environmental management within a One Health perspective on pharmaceutical contamination in aquatic systems.

## Figures and Tables

**Table 1:** Age groups defined according to EPA guidelines and their respective body weight (kg) and daily water intake (DWI, L/day) values adopted for human health risk assessment.

Age Range (Years)	Body Weight (kg)	DWI (L/Day)
0–1	7.4	0.97
1–10	21.3	0.52
10–20	51.8	1.64
20–30	70.8	2.85
30–40	72.6	2.97
40–50	74.2	2.97
50–60	75.9	2.98
60–70	75.1	2.97
70–80	72.4	2.27
80+	68.7	2.12

Note: Age groups were defined based on EPA guidelines [22]. Body weight (50th percentile) and daily drinking water intake (DWI) values were both obtained from the EPA Exposure Factors Handbook [22].

**Table 2:** Concentration of pharmaceutical compounds identified in consumption water in Portugal in ng/L.

Matrix	Therapeutic Classes	Pharmaceuticals	n	Min	Mean	Max	Reference
Bottled water	Anti-inflammatory	Diclofenac	2	3.95	5.81	7.66	[49]
	Anti-inflammatory	Salicylic acid	2	21.20	25.90	30.60	[50]
	Antibiotic	Erythromycin	2	0.50	3.10	5.69	[25]
	Antibiotic	Sulfadiazine	2	0.40	0.70	1.00	[25]
	Antibiotic	Sulfapyridine	2	1.00	1.50	2.00	[25]
	Anticoagulant	Warfarin	2	4.07	7.64	11.20	[49]
	Anticonvulsant	Carbamazepine	3	1.90	12.67	22.10	[25,50]
	Cardiovascular	Atenolol	2	0.21	0.61	1.00	[49]
	Pesticide	Chlorfenvinphos	2	0.49	2.19	3.89	[25]
Tap water	Anti-inflammatory	Diclofenac	1	7.87	7.87	7.87	[49]
	Anti-inflammatory	Salicylic acid	2	39.40	52.70	66.00	[50]
	Anticoagulant	Warfarin	2	0.39	2.14	3.89	[49]
	Anticonvulsant	Carbamazepine	3	3.34	15.21	22.30	[49,50]
	Antidepressant	Fluoxetine	2	0.27	1.09	1.90	[50]
	Illicit drug	Cocaine	3	40.00	166.33	340.00	[51]
	Metabolite	Benzoylecgonine	1	104.00	104.00	104.00	[51]
	Pesticide	Chlorfenvinphos	2	2.46	4.48	6.50	[49]
Surface water	Analgesic	Acetaminophen	77	0.10	256.11	10,587.00	[52,53]
	Analgesic	Codeine	3	1.50	1.80	2.10	[53]
	Analgesic	Tramadol	4	8.70	1625.63	4444.00	[53,54]
	Anti-inflammatory	Diclofenac	31	0.99	162.04	3165.00	[52,53,55,56,57,58]
	Anti-inflammatory	Ibuprofen	56	1.38	321.89	3774.00	[52,53,59]
	Anti-inflammatory	Ketoprofen	23	7.90	33.25	75.30	[52,53,57,58,60]
	Anti-inflammatory	Naproxen	16	12.30	176.28	1266.00	[52,53,57,58]
	Anti-inflammatory	Nimesulide	1	6.50	6.50	6.50	[52]
	Anti-inflammatory	Phenylbutazone	3	132.70	132.70	132.70	[61]
	Antibiotic	Amoxicillin	3	59.00	6416.73	15,382.00	[53]
	Antibiotic	Azithromycin	17	6.20	268.41	2819.00	[52,55,57,58,62]
	Antibiotic	Ciprofloxacin	5	59.30	137.16	339.00	[57,58,62,63]
	Antibiotic	Clarithromycin	31	0.29	30.37	269.00	[52,53,55,56,57,62]
	Antibiotic	Enrofloxacin	2	67.00	84.75	102.50	[63]
	Antibiotic	Erythromycin	10	0.06	11.37	38.80	[55,56]
	Antibiotic	Isoniazid	3	3.30	5.87	8.40	[53]
	Antibiotic	Lincomycin	16	0.23	0.38	0.70	[56]
	Antibiotic	Ofloxacin	1	120.00	120.00	120.00	[57,58,62,63]
	Antibiotic	Roxithromycin	3	0.08	0.13	0.20	[56]
	Antibiotic	Sulfadiazine	1	114.00	114.00	114.00	[52]
	Antibiotic	Sulfadimethoxine	19	0.04	0.88	8.40	[53,56]
	Antibiotic	Sulfamethazine	9	4.87	49.75	123.00	[52,57]
	Antibiotic	Sulfamethoxazole	22	0.44	9.97	53.30	[52,56,57,64]
	Antibiotic	Sulfapyridine	2	11.60	13.40	15.20	[52]
	Antibiotic	Tetracycline	1	55.10	55.10	55.10	[52]
	Antibiotic	Tiamulin	6	0.02	0.06	0.10	[56]
	Antibiotic	Trimethoprim	6	3.89	43.30	110.00	[52,62,64]
	Anticonvulsant	Carbamazepine	92	0.37	52.70	354.00	[50,52,53,56,57,62,64,65]
	Anticonvulsant	Primidone	16	1.14	3.88	13.50	[56]
	Anticonvulsant	Topiramate	7	0.66	84.39	237.00	[52]
	Antidepressant	Bupropion	4	15.70	32.98	60.60	[52]
	Antidepressant	Citalopram	11	1.67	30.72	67.90	[52,55,57,62]
	Antidepressant	Fluoxetine	69	1.90	5.67	28.90	[50,52,54,55,62]
	Antidepressant	Paroxetine	3	25.50	25.53	25.60	[57]
	Antidepressant	Sertraline	6	5.40	13.45	23.30	[52,55,62]
	Antidepressant	Trazodone	9	2.15	31.88	148.00	[52,57]
	Antidepressant	Venlafaxine	19	3.30	89.57	641.00	[52,53,57,62]
	Antifungal	Fluconazole	4	227.50	355.90	573.80	[61]
	Antihistamine	Cetirizine	1	40.00	40.00	40.00	[65]
	Benzodiazepine	Diazepam	2	3.65	8.78	13.90	[52,64]
	Benzodiazepine	Lorazepam	4	21.10	34.33	49.10	[58]
	Beta-blocker	Atenolol	3	3.40	820.93	2370.00	[53]
	Beta-blocker	Bisoprolol	11	4.50	335.55	2360.00	[53,58]
	Cardiovascular	Diltiazem	1	25.60	25.60	25.60	[52]
	Cardiovascular	Metformin	3	35.99	35.99	35.99	[61]
	Cardiovascular	Propranolol	20	0.03	60.40	1159.00	[53,56,64]
	Cardiovascular	Ramipril	3	2.70	913.07	2659.00	[53]
	Cardiovascular	Simvastatin	1	42.90	42.90	42.90	[58]
	Cardiovascular	Sotalol	3	5.20	8.27	11.50	[53]
	Cardiovascular	Warfarin	1	2.20	2.20	2.20	[53]
	Contrast agent	Iohexol	2	10.10	40.30	70.50	[56]
	Contrast agent	Iomeprol	16	5.24	64.51	386.00	[56]
	Contrast agent	Iopamidol	1	4.31	4.31	4.31	[56]
	Contrast agent	Iopromide	16	43.10	461.20	2810.00	[56]
	Corticosteroid	Betamethasone	4	20.00	274.70	701.00	[61]
	Corticosteroid	Prednisone	3	36.80	41.27	50.20	[61]
	Diuretic	Furosemide	10	66.70	1271.19	8216.00	[53,58]
	Diuretic	Hydrochlorothiazide	6	31.00	185.42	389.00	[58,60]
	Hormone	17-alpha-Ethinylestradiol	11	0.50	4.97	20.40	[66,67,68]
	Hormone	17-beta-Estradiol	11	1.60	5.72	12.05	[66,67,68]
	Hormone	Estrone	10	1.40	6.34	10.40	[67,68]
	Lipid-lowering	Atorvastatin	4	12.10	45.93	68.40	[52,53]
	Lipid-lowering	Bezafibrate	23	0.07	95.32	770.00	[55,56,58]
	Lipid-lowering	Clofibric	3	8.90	403.33	1165.00	[53]
	Lipid-lowering	Fenofibrate acid	3	1.48	29.53	70.30	[64]
	Lipid-lowering	Gemfibrozil	14	5.72	33.39	91.20	[52,55,58]
	Metabolite	10,11-Epoxycarbamazepine	10	33.20	34.62	40.40	[52,57]
	Metabolite	Benzoylecgonine	1	72.40	72.40	72.40	[54]
	Metabolite	Carboxyibuprofen	3	43.80	459.93	1227.00	[52]
	Metabolite	Citalopram propionic acid	4	9.30	15.95	24.00	[52]
	Metabolite	Hydroxyibuprofen	23	15.30	239.30	1673.00	[52,57]
	Metabolite	p-Aminophenol	4	400.00	950.00	1630.00	[69]
	Metabolite	Paracetamol-glucuronide	3	180.00	1370.00	3570.00	[69]
	Metabolite	Salicylic acid	67	25.00	109.72	348.00	[50,52]
	Proton pump inhibitor	Omeprazole	3	11.10	3223.03	8255.00	[53]

Note: *n* = the number of analytical determinations.

**Table 3:** Average concentration (ng/L) of pharmaceutical compounds detected in different water matrices in Portugal and comparative concentration ratios across matrices.

Pharmaceutical	SW	TW	BW	AF	Observations	Reference
Carbamazepine	52.70	15.21	12.67	3.5	Moderate persistence across all matrices	[25,49,50,52,53,56,57,62,64,65]
Diclofenac	162.04	7.87	5.81	20.6	Markedly lower levels in consumption waters; Low persistence	[49,52,53,55,56,57,58]
Salicylic acid	109.72	52.70	25.90	2.1	Recurrent and less attenuated; Moderate pseudo-persistence	[50,52]
Warfarin	2.20	2.14	7.64	1.0	Higher in bottled water	[49,53]
Sulfadiazine	114.00	–	0.70	–	Large contrast between surface water and bottled water; Low persistence	[25,52]
Sulfapyridine	14.40	–	1.50	–	Low persistence	[25,52]
Erythromycin	11.37	–	3.10	–	Moderate persistence	[25,55,56]
Atenolol	820.93	–	0.61	–	Large contrast between surface water and bottled water; Low persistence	[25,53]

Note: SW = surface water; TW = tap water; BW = bottled water. AF = Across-Matrix Factor, concentration ratio between surface water and tap water (SW/TP); high values indicate greater persistence or possible recontamination.

**Table 4:** Risk classification of pharmaceutical compounds identified in surface water based on their highest detected concentration.

Risk Classification	RQ	Pharmaceutical
Uncategorized	Not assessed	Cocaine and benzoylecgonine
High	>1.0	Ramipril, 17-alpha-Ethinylestradiol, Betamethasone, Citalopram, Lorazepam, Furosemide, Omeprazole, 17-beta-Estradiol, Estrone, Amoxicillin and Citalopram propionic acid
Moderate	0.1–1.0	Diclofenac, Trazodone, Acetaminophen, Atenolol, Propranolol, Hydrochlorothiazide, Bisoprolol, Prednisone, Carbamazepine, Paracetamol-glucuronide and Azithromycin
Low	<0.1	Iopamidol, Tiamulin, Iohexol, Lincomycin, Fenofibrate acid, Roxithromycin, Iomeprol, Phenylbutazone, Codeine, Bupropion, Sulfadimethoxine, Tetracycline, Isoniazid, Nimesulide, Topiramate, Sulfapyridine, Iopromide, Metformin, Gemfibrozil, Sotalol, Paroxetine, Fluoxetine, Sulfadiazine, Diltiazem, Sulfamethazine, Warfarin, Diazepam, 10,11-Epoxycarbamazepine, Sulfamethoxazole, Enrofloxacin, Primidone, Ofloxacin, Erythromycin, Clarithromycin, Salicylic acid, Trimethoprim, Ketoprofen, Bezafibrate, Carboxyibuprofen, Clofibric, Venlafaxine, Hydroxyibuprofen, Naproxen, Ibuprofen, Atorvastatin, Cetirizine, p-Aminophenol, Simvastatin, Tramadol, Sertraline, Ciprofloxacin and Fluconazole

**Table 5:** Assessment of the priority of concern of pharmaceutical compounds (RQ_screen) identified in bottled water regarding human health in different age groups.

Pharmaceutical Compounds	Age Ranges (Years)									
	0–1	1–10	10–20	20–30	30–40	40–50	50–60	60–70	70–80	80+
Potential risk to human health considering the best-case scenario (minimum concentration)										
Atenolol	0.033	0.006	0.008	0.010	0.010	0.010	0.010	0.010	0.008	0.008
Carbamazepine	0.797	0.148	0.192	0.245	0.249	0.243	0.239	0.240	0.191	0.188
Chlorfenvinphos	0.002	0.000	0.001	0.001	0.001	0.001	0.001	0.001	0.001	0.001
Diclofenac	0.994	0.185	0.240	0.305	0.310	0.304	0.298	0.300	0.238	0.234
Erythromycin	0.052	0.010	0.013	0.016	0.016	0.016	0.016	0.016	0.013	0.012
Salicylic acid	0.321	0.060	0.078	0.099	0.100	0.098	0.096	0.097	0.077	0.076
Sulfamethazine	0.005	0.001	0.001	0.002	0.002	0.002	0.002	0.002	0.001	0.001
Sulfapyridine	0.026	0.005	0.006	0.008	0.008	0.008	0.008	0.008	0.006	0.006
Warfarin	3.201	0.596	0.773	0.983	0.999	0.977	0.959	0.966	0.766	0.754
Potential risk to human health considering the real-case scenario (mean concentration)										
Atenolol	0.095	0.018	0.023	0.029	0.030	0.029	0.029	0.029	0.023	0.022
Carbamazepine	5.315	0.990	1.284	1.632	1.659	1.623	1.592	1.603	1.271	1.251
Chlorfenvinphos	0.010	0.002	0.002	0.003	0.003	0.003	0.003	0.003	0.002	0.002
Diclofenac	1.461	0.272	0.353	0.449	0.456	0.446	0.438	0.441	0.349	0.344
Erythromycin	0.325	0.060	0.078	0.100	0.101	0.099	0.097	0.098	0.078	0.076
Salicylic acid	0.393	0.073	0.095	0.121	0.123	0.120	0.118	0.118	0.094	0.092
Sulfamethazine	0.009	0.002	0.002	0.003	0.003	0.003	0.003	0.003	0.002	0.002
Sulfapyridine	0.039	0.007	0.009	0.012	0.012	0.012	0.012	0.012	0.009	0.009
Warfarin	6.005	1.118	1.450	1.844	1.874	1.834	1.799	1.812	1.436	1.414
Potential risk to human health considering the worst-case scenario (maximum concentration)										
Atenolol	0.157	0.029	0.038	0.048	0.049	0.048	0.047	0.047	0.038	0.037
Carbamazepine	9.270	1.726	2.239	2.847	2.893	2.831	2.777	2.797	2.217	2.182
Chlorfenvinphos	0.018	0.003	0.004	0.005	0.006	0.005	0.005	0.005	0.004	0.004
Diclofenac	1.928	0.359	0.466	0.592	0.602	0.589	0.577	0.582	0.461	0.454
Erythromycin	0.597	0.111	0.144	0.183	0.186	0.182	0.179	0.180	0.143	0.140
Salicylic acid	0.464	0.086	0.112	0.142	0.145	0.142	0.139	0.140	0.111	0.109
Sulfamethazine	0.013	0.002	0.003	0.004	0.004	0.004	0.004	0.004	0.003	0.003
Sulfapyridine	0.052	0.010	0.013	0.016	0.016	0.016	0.016	0.016	0.013	0.012
Warfarin	8.809	1.641	2.128	2.705	2.749	2.690	2.638	2.658	2.107	2.074

Note: Cells highlighted in green (X) indicate that, for the age range evaluated, the pharmaceutical compound was classified as low priority (RQ_screen < 0.01). Cells highlighted in yellow (X) correspond to compounds classified as moderate priority (0.01 ≤ RQ_screen < 0.1). Cells highlighted in orange (X) indicate high priority (0.1 ≤ RQ_screen < 1.0), while cells highlighted in red (X) identify compounds classified as critical-priority, with RQ_screen ≥ 1.0, for the age range considered.

**Table 6:** Assessment of the priority of concern of pharmaceutical compounds (RQ_screen) identified in tap water regarding human health in different age groups.

Pharmaceutical Compounds	Age Ranges (Years)									
	0–1	1–10	10–20	20–30	30–40	40–50	50–60	60–70	70–80	80+
Potential risk to human health considering the best-case scenario (minimum concentration)										
Carbamazepine	1.401	0.261	0.338	0.430	0.437	0.428	0.420	0.423	0.335	0.330
Chlorfenvinphos	0.011	0.002	0.003	0.003	0.004	0.003	0.003	0.003	0.003	0.003
Diclofenac	1.981	0.369	0.478	0.608	0.618	0.605	0.593	0.598	0.474	0.466
Fluoxetine	0.012	0.002	0.003	0.004	0.004	0.004	0.004	0.004	0.003	0.003
Salicylic acid	0.597	0.111	0.144	0.183	0.186	0.182	0.179	0.180	0.143	0.141
Warfarin	0.307	0.057	0.074	0.094	0.096	0.094	0.092	0.093	0.073	0.072
Potential risk to human health considering the real-case scenario (mean concentration)										
Carbamazepine	6.380	1.188	1.541	1.959	1.991	1.948	1.911	1.925	1.526	1.502
Chlorfenvinphos	0.021	0.004	0.005	0.006	0.006	0.006	0.006	0.006	0.005	0.005
Diclofenac	1.981	0.369	0.478	0.608	0.618	0.605	0.593	0.598	0.474	0.466
Fluoxetine	0.047	0.009	0.011	0.015	0.015	0.014	0.014	0.014	0.011	0.011
Salicylic acid	0.799	0.149	0.193	0.245	0.249	0.244	0.239	0.241	0.191	0.188
Warfarin	1.683	0.313	0.407	0.517	0.525	0.514	0.504	0.508	0.403	0.396
Potential risk to human health considering the worst-case scenario (maximum concentration)										
Carbamazepine	9.354	1.742	2.259	2.873	2.919	2.856	2.802	2.822	2.237	2.202
Chlorfenvinphos	0.030	0.006	0.007	0.009	0.009	0.009	0.009	0.009	0.007	0.007
Diclofenac	1.981	0.369	0.478	0.608	0.618	0.605	0.593	0.598	0.474	0.466
Fluoxetine	0.082	0.015	0.020	0.025	0.026	0.025	0.025	0.025	0.020	0.019
Salicylic acid	1.001	0.186	0.242	0.307	0.312	0.306	0.300	0.302	0.239	0.236
Warfarin	3.059	0.570	0.739	0.940	0.955	0.934	0.916	0.923	0.732	0.720

Note: Cells highlighted in green (X) indicate that, for the age range evaluated, the pharmaceutical compound was classified as low priority (RQ_screen < 0.01). Cells highlighted in yellow (X) correspond to compounds classified as moderate priority (0.01 ≤ RQ_screen < 0.1). Cells highlighted in orange (X) indicate high priority (0.1 ≤ RQ_screen < 1.0), while cells highlighted in red (X) identify compounds classified as critical priority, with RQ_screen ≥ 1.0, for the age range considered.

**Table 7:** Classification of pharmaceuticals according to the Risk Quality Screening Index (RQ_screen) and definition of concern priorities considering the average concentration value detected in surface waters of Portugal (from Table 2).

RQ_screen	Priority	n	Pharmaceuticals
<0.01	Low	8	Fenofibrate acid, Iohexol, Iomeprol, Iopamidol, Lincomycin, Roxithromycin, Sulfadimethoxine and Tiamulin
0.01–0.1	Moderate	11	Bupropion, Codeine, Fluoxetine, Gemfibrozil, Iopromide, Isoniazid, Nimesulide, Phenylbutazone, Sulfamethoxazole, Tetracycline and Topiramate
0.1–1.0	High	22	10,11-Epoxycarbamazepine, Bezafibrate, Clarithromycin, Diazepam, Diltiazem, Enrofloxacin, Erythromycin, Hydroxyibuprofen, Ibuprofen, Metformin, Naproxen, Ofloxacin, Paroxetine, Primidone, Salicylic acid, Sotalol, Sulfadiazine, Sulfamethazine, Sulfapyridine, Trimethoprim, Venlafaxine and Warfarin
>1.0	Critical	33	17-alpha-Ethinylestradiol, 17-beta-Estradiol, Acetaminophen, Amoxicillin, Atenolol, Atorvastatin, Azithromycin, Betamethasone, Bisoprolol, Carbamazepine, Carboxyibuprofen, Cetirizine, Ciprofloxacin, Citalopram, Citalopram propionic acid, Clofibric, Diclofenac, Estrone, Fluconazole, Furosemide, Hydrochlorothiazide, Ketoprofen, Lorazepam, Omeprazole, p-Aminophenol, Paracetamol-glucuronide, Prednisone, Propranolol, Ramipril, Sertraline, Simvastatin, Tramadol and Trazodone

**Table 8:** Characteristics and relevance of priority pharmaceutical residues for monitoring in Portugal.

Pharmaceutical	CAS Number	Therapeutic Class	WS (mg/L)	logKow	C_SW (ng/L)	Removal in WWTP	Persistence	Main Environmental Concern
Ciprofloxacin	85721-33-1	Antibiotic	1350.0	−0.57	5.93–339.0	>70%	High	Antimicrobial resistance
Fluconazole	86386-73-4	Antifungal	1390.0	0.58	227.5–573.8	>70%	High	Persistence; Chronic toxicity
Propranolol	525-66-6	Beta-blocker	79.4	3.03	0.03–1159.0	30–70%	Moderate	Effects on aquatic organisms
Estradiol (E2)	50-28-2	Hormone	6.8	3.63	0.50–20.40	30–70%	High	Endocrine disruption
Estrone (E1)	53-16-7	Hormone	3.9	4.02	1.40–10.40	<30%	High	Endocrine disruption
Diclofenac	15307-86-5	NSAIDs	4.5	4.98	0.99–3165.0	<30%	High	Effects on aquatic organisms
Carbamazepine	298-46-4	Anticonvulsant	152.0	2.77	0.37–354.0	30–70%	Very high	High persistence

Note: NSAIDs = Nonsteroidal anti-inflammatory drugs; C_SW = surface water concentration; WS = Water solubility; WWTP = Wastewater Treatment Plants.

## Data Availability

No new data were created or analyzed in this study. All data reported are from publicly available sources cited in the References.

## Supplementary Materials

The following supporting information can be downloaded at: https://www.mdpi.com/article/10.3390/ijerph23070838/s1, Table S1: Database of pharmaceutical residues present in surface, tap and bottled water in Portugal; Table S2: Database of acceptable daily intake values for each pharmaceutical residue evaluated in this study.

## Abbreviations

The following abbreviations are used in this manuscript:

ADI	Acceptable Daily Intake
AF	Across-Matrix Factor
BW	Body weight
BW	Bottled water
C_SW	Surface water concentration
DWEL	Drinking Water Equivalent Level
DWI	Drinking water intake
EPA	Environmental Protection Agency
FOE	Frequency of exposure
HQ	Hazard Quotient
RD	Reference dose
RQ	Risk quotient
RQ_screen	Screening risk quotient
SDG	Sustainable Development Goal
SW	Surface water
*t*_1/2_	Half-life
TW	Tap water
UF	Uncertainty factor
WEL	Water Equivalent Level
WS	Water solubility
WWTPs	Wastewater Treatment Plants
