# Supplementary material for: Incorporating a Screening-Level Risk Quotient (RQ_screen) for Assessing Human Health Risk of Pharmaceutical Residues in Consumption Water
Source: Int J Environ Res Public Health. 2026 Jun 25;23(7):838. doi: 10.3390/ijerph23070838 (PMC13409792; doi:10.3390/ijerph23070838)
Supplement: Supplementary file 1 [file ijerph-23-00838-s001.zip › ijerph-4348707-supplementary.pdf]

Incorporating a Screening-Level Risk Quotient (RQ_screen) for Assessing Human Health Risk of Pharmaceutical Residues in Consumption Water

Gabriel Souza-Silva [a], Igor F.C. dos Santos [b], Inês B. Gomes [c], Manuel Simões [c], Micheline R. Silveira [a]; Vítor J.P. Vilar [d]; Ana I. Gomes [d]*

[a] Faculty of Pharmacy, Federal University of Minas Gerais, 31270-901 Minas Gerais, Brazil.

[b] Sergio Arouca National School of Public Health, Oswaldo Cruz Foundation, 21041-210 Rio de Janeiro, Brazil.

[c] LEPABE, ALiCE, Faculty of Engineering, University of Porto, 4200-465 Porto, Portugal

[d] LSRE-LCM, ALiCE, Faculty of Engineering, University of Porto, 4200-465 Porto, Portugal

* Corresponding author(s): anaisa@fe.up.pt

Table S1. Database of pharmaceutical residues present in surface, tap and bottled water in Portugal.

Name of Analyte	CAS number	Therapeutic Group	Matrix	Sampling	MEC	Unit	Reference
10,11-Epoxycarbamazepine	36507-30-9	Antiepileptic	Surface Water	2013	33,2	ng/L	[1]
10,11-Epoxycarbamazepine	36507-30-9	Antiepileptic	Surface Water	2014	33,3	ng/L	[1]
10,11-Epoxycarbamazepine	36507-30-9	Antiepileptic	Surface Water	2014	33,5	ng/L	[1]
10,11-Epoxycarbamazepine	36507-30-9	Antiepileptic	Surface Water	2013	33,6	ng/L	[2]
10,11-Epoxycarbamazepine	36507-30-9	Antiepileptic	Surface Water	2014	33,7	ng/L	[1]
10,11-Epoxycarbamazepine	36507-30-9	Antiepileptic	Surface Water	2014	33,8	ng/L	[1]
10,11-Epoxycarbamazepine	36507-30-9	Antiepileptic	Surface Water	2014	34,1	ng/L	[1]
10,11-Epoxycarbamazepine	36507-30-9	Antiepileptic	Surface Water	2014	34,3	ng/L	[1]
10,11-Epoxycarbamazepine	36507-30-9	Antiepileptic	Surface Water	2013	36,3	ng/L	[1]
10,11-Epoxycarbamazepine	36507-30-9	Antiepileptic	Surface Water	2013	40,4	ng/L	[1]
17-alpha-Ethinylestradiol	57-63-6	Estrogen	Surface Water	2011	11	ng/L	[3]
17-alpha-Ethinylestradiol	57-63-6	Estrogen	Surface Water	2009	3	ng/L	[4]
17-alpha-Ethinylestradiol	57-63-6	Estrogen	Surface Water	2009	3,4	ng/L	[4]
17-alpha-Ethinylestradiol	57-63-6	Estrogen	Surface Water	2009	3,8	ng/L	[4]
17-alpha-Ethinylestradiol	57-63-6	Estrogen	Surface Water	2009	3,1	ng/L	[4]
17-alpha-Ethinylestradiol	57-63-6	Estrogen	Surface Water	2009	4,4	ng/L	[4]
17-alpha-Ethinylestradiol	57-63-6	Estrogen	Surface Water	2009	2,3	ng/L	[4]
17-alpha-Ethinylestradiol	57-63-6	Estrogen	Surface Water	2010	20,4	ng/L	[5]
17-alpha-Ethinylestradiol	57-63-6	Estrogen	Surface Water	2010	1,2	ng/L	[5]
17-alpha-Ethinylestradiol	57-63-6	Estrogen	Surface Water	2010	0,5	ng/L	[5]
17-alpha-Ethinylestradiol	57-63-6	Estrogen	Surface Water	2010	1,6	ng/L	[5]
17-beta-Estradiol	50-28-2	Estrogen	Surface Water	2011	12,05	ng/L	[3]
17-beta-Estradiol	50-28-2	Estrogen	Surface Water	2009	5,9	ng/L	[4]
17-beta-Estradiol	50-28-2	Estrogen	Surface Water	2009	4,8	ng/L	[4]
17-beta-Estradiol	50-28-2	Estrogen	Surface Water	2009	5,4	ng/L	[4]
17-beta-Estradiol	50-28-2	Estrogen	Surface Water	2009	5,5	ng/L	[4]
17-beta-Estradiol	50-28-2	Estrogen	Surface Water	2009	5,7	ng/L	[4]
17-beta-Estradiol	50-28-2	Estrogen	Surface Water	2009	5,5	ng/L	[4]
17-beta-Estradiol	50-28-2	Estrogen	Surface Water	2010	5,2	ng/L	[5]
17-beta-Estradiol	50-28-2	Estrogen	Surface Water	2010	5,9	ng/L	[5]
17-beta-Estradiol	50-28-2	Estrogen	Surface Water	2010	1,6	ng/L	[5]
17-beta-Estradiol	50-28-2	Estrogen	Surface Water	2010	5,4	ng/L	[5]
Acetaminophen	103-90-2	Analgesics	Surface Water	2013	4,9	ng/L	[1]

Acetaminophen	103-90-2	Analgesics	Surface Water	2014	19	ng/L	[1]
Acetaminophen	103-90-2	Analgesics	Surface Water	2014	19,7	ng/L	[1]
Acetaminophen	103-90-2	Analgesics	Surface Water	2018	20,6	ng/L	[1]
Acetaminophen	103-90-2	Analgesics	Surface Water	2013	22,3	ng/L	[1]
Acetaminophen	103-90-2	Analgesics	Surface Water	2014	22,7	ng/L	[1]
Acetaminophen	103-90-2	Analgesics	Surface Water	2013	33,6	ng/L	[1]
Acetaminophen	103-90-2	Analgesics	Surface Water	2013	33,6	ng/L	[1]
Acetaminophen	103-90-2	Analgesics	Surface Water	2013	35,1	ng/L	[1]
Acetaminophen	103-90-2	Analgesics	Surface Water	2019	35,2	ng/L	[1]
Acetaminophen	103-90-2	Analgesics	Surface Water	2014	38,3	ng/L	[1]
Acetaminophen	103-90-2	Analgesics	Surface Water	2014	40,5	ng/L	[1]
Acetaminophen	103-90-2	Analgesics	Surface Water	2013	43,6	ng/L	[1]
Acetaminophen	103-90-2	Analgesics	Surface Water	2019	43,7	ng/L	[1]
Acetaminophen	103-90-2	Analgesics	Surface Water	2019	44,3	ng/L	[1]
Acetaminophen	103-90-2	Analgesics	Surface Water	2013	47	ng/L	[1]
Acetaminophen	103-90-2	Analgesics	Surface Water	2014	48	ng/L	[1]
Acetaminophen	103-90-2	Analgesics	Surface Water	2013	49	ng/L	[1]
Acetaminophen	103-90-2	Analgesics	Surface Water	2013	51,1	ng/L	[1]
Acetaminophen	103-90-2	Analgesics	Surface Water	2019	51,7	ng/L	[1]
Acetaminophen	103-90-2	Analgesics	Surface Water	2014	59,2	ng/L	[1]
Acetaminophen	103-90-2	Analgesics	Surface Water	2014	71,2	ng/L	[1]
Acetaminophen	103-90-2	Analgesics	Surface Water	2014	72,3	ng/L	[1]
Acetaminophen	103-90-2	Analgesics	Surface Water	2014	74	ng/L	[1]
Acetaminophen	103-90-2	Analgesics	Surface Water	2013	75,4	ng/L	[1]
Acetaminophen	103-90-2	Analgesics	Surface Water	2014	76,6	ng/L	[1]
Acetaminophen	103-90-2	Analgesics	Surface Water	2018	77,6	ng/L	[1]
Acetaminophen	103-90-2	Analgesics	Surface Water	2018	93,8	ng/L	[1]
Acetaminophen	103-90-2	Analgesics	Surface Water	2013	95,5	ng/L	[1]
Acetaminophen	103-90-2	Analgesics	Surface Water	2014	99	ng/L	[1]
Acetaminophen	103-90-2	Analgesics	Surface Water	2014	120	ng/L	[1]
Acetaminophen	103-90-2	Analgesics	Surface Water	2013	133	ng/L	[1]
Acetaminophen	103-90-2	Analgesics	Surface Water	2014	138	ng/L	[1]
Acetaminophen	103-90-2	Analgesics	Surface Water	2014	138	ng/L	[1]
Acetaminophen	103-90-2	Analgesics	Surface Water	2014	140	ng/L	[1]
Acetaminophen	103-90-2	Analgesics	Surface Water	2018	142	ng/L	[1]
Acetaminophen	103-90-2	Analgesics	Surface Water	2013	144	ng/L	[1]
Acetaminophen	103-90-2	Analgesics	Surface Water	2019	148	ng/L	[1]
Acetaminophen	103-90-2	Analgesics	Surface Water	2014	151	ng/L	[1]
Acetaminophen	103-90-2	Analgesics	Surface Water	2013	170	ng/L	[1]
Acetaminophen	103-90-2	Analgesics	Surface Water	2014	175	ng/L	[1]
Acetaminophen	103-90-2	Analgesics	Surface Water	2014	327	ng/L	[1]
Acetaminophen	103-90-2	Analgesics	Surface Water	2014	527	ng/L	[1]
Acetaminophen	103-90-2	Analgesics	Surface Water	2024	8,7	ng/L	[6]
Acetaminophen	103-90-2	Analgesics	Surface Water	2024	1450	ng/L	[6]
Acetaminophen	103-90-2	Analgesics	Surface Water	2024	10587	ng/L	[6]
Amoxicillin	26787-78-0	Antibiotics	Surface Water	2024	59,2	ng/L	[6]
Amoxicillin	26787-78-0	Antibiotics	Surface Water	2024	3809	ng/L	[6]
Amoxicillin	26787-78-0	Antibiotics	Surface Water	2024	15382	ng/L	[6]
Atenolol	29122-68-7	Beta-blocker	Surface Water	2024	3,4	ng/L	[6]
Atenolol	29122-68-7	Beta-blocker	Surface Water	2024	89,4	ng/L	[6]

Atenolol	29122-68-7	Beta-blocker	Surface Water	2024	2370	ng/L	[6]
Atenolol	29122-68-7	Beta-blocker	Bottled water	2013	0,21	ng/L	[7]
Atenolol	29122-68-7	Beta-blocker	Bottled water	2013	1	ng/L	[7]
Atorvastatin	134523-00-5	Statine	Surface Water	2024	12,1	ng/L	[6]
Atorvastatin	134523-00-5	Statine	Surface Water	2024	34,9	ng/L	[6]
Atorvastatin	134523-00-5	Statine	Surface Water	2024	68,4	ng/L	[6]
Atorvastatin	134523-00-5	Statine	Surface Water	2019	68,3	ng/L	[1]
Azithromycin	83905-01-5	Antibiotics	Surface Water	2017	2819	ng/L	[8]
Azithromycin	83905-01-5	Antibiotics	Surface Water	2017	221	ng/L	[8]
Azithromycin	83905-01-5	Antibiotics	Surface Water	2014	35,66	ng/L	[9]
Azithromycin	83905-01-5	Antibiotics	Surface Water	2014	33,91	ng/L	[9]
Azithromycin	83905-01-5	Antibiotics	Surface Water	2014	32,15	ng/L	[9]
Azithromycin	83905-01-5	Antibiotics	Surface Water	2019	6,2	ng/L	[1]
Azithromycin	83905-01-5	Antibiotics	Surface Water	2013	11,1	ng/L	[2]
Azithromycin	83905-01-5	Antibiotics	Surface Water	2013	15	ng/L	[2]
Azithromycin	83905-01-5	Antibiotics	Surface Water	2013	16,2	ng/L	[1]
Azithromycin	83905-01-5	Antibiotics	Surface Water	2019	41,7	ng/L	[1]
Azithromycin	83905-01-5	Antibiotics	Surface Water	2019	73	ng/L	[1]
Azithromycin	83905-01-5	Antibiotics	Surface Water	2018	187	ng/L	[1]
Azithromycin	83905-01-5	Antibiotics	Surface Water	2018	532	ng/L	[1]
Azithromycin	83905-01-5	Antibiotics	Surface Water	2009	136	ng/L	[10]
Azithromycin	83905-01-5	Antibiotics	Surface Water	2009	163	ng/L	[10]
Azithromycin	83905-01-5	Antibiotics	Surface Water	2009	147	ng/L	[10]
Azithromycin	83905-01-5	Antibiotics	Surface Water	2009	94,7	ng/L	[10]
Benzoylecgonine	519-09-5	Drug	Tap Water	2020	104	ng/L	[11]
Benzoylecgonine	519-09-5	Metacolic	Surface Water	2018	72,4	ng/L	[12]
Betamethasone	378-44-9	Glucocorticoids	Surface Water	2016	20	ng/L	[13]
Betamethasone	378-44-9	Glucocorticoids	Surface Water	2016	701	ng/L	[13]
Betamethasone	378-44-9	Glucocorticoids	Surface Water	2016	246,2	ng/L	[13]
Betamethasone	378-44-9	Glucocorticoids	Surface Water	2016	131,6	ng/L	[13]
Bezafibrate	41859-67-0	Lipid-lowering	Surface Water	2018	0,1	ng/L	[14]
Bezafibrate	41859-67-0	Lipid-lowering	Surface Water	2018	0,09	ng/L	[14]
Bezafibrate	41859-67-0	Lipid-lowering	Surface Water	2018	0,07	ng/L	[14]
Bezafibrate	41859-67-0	Lipid-lowering	Surface Water	2018	0,09	ng/L	[14]
Bezafibrate	41859-67-0	Lipid-lowering	Surface Water	2018	0,09	ng/L	[14]
Bezafibrate	41859-67-0	Lipid-lowering	Surface Water	2018	0,07	ng/L	[14]
Bezafibrate	41859-67-0	Lipid-lowering	Surface Water	2018	10,2	ng/L	[14]
Bezafibrate	41859-67-0	Lipid-lowering	Surface Water	2018	3,95	ng/L	[14]
Bezafibrate	41859-67-0	Lipid-lowering	Surface Water	2018	3,94	ng/L	[14]
Bezafibrate	41859-67-0	Lipid-lowering	Surface Water	2018	3,23	ng/L	[14]
Bezafibrate	41859-67-0	Lipid-lowering	Surface Water	2018	1,35	ng/L	[14]
Bezafibrate	41859-67-0	Lipid-lowering	Surface Water	2018	0,64	ng/L	[14]
Bezafibrate	41859-67-0	Lipid-lowering	Surface Water	2009	770	ng/L	[10]
Bezafibrate	41859-67-0	Lipid-lowering	Surface Water	2009	19,1	ng/L	[10]
Bezafibrate	41859-67-0	Lipid-lowering	Surface Water	2009	706	ng/L	[10]
Bezafibrate	41859-67-0	Lipid-lowering	Surface Water	2009	10,9	ng/L	[10]
Bezafibrate	41859-67-0	Lipid-lowering	Surface Water	2009	334	ng/L	[10]
Bezafibrate	41859-67-0	Lipid-lowering	Surface Water	2009	21,2	ng/L	[10]
Bezafibrate	41859-67-0	Lipid-lowering	Surface Water	2009	256	ng/L	[10]
Bezafibrate	41859-67-0	Lipid-lowering	Surface Water	2009	10,2	ng/L	[10]

Bezafibrate	41859-67-0	Lipid-lowering	Surface Water	2014	15,5	ng/L	[9]
Bezafibrate	41859-67-0	Lipid-lowering	Surface Water	2014	13,69	ng/L	[9]
Bezafibrate	41859-67-0	Lipid-lowering	Surface Water	2014	11,86	ng/L	[9]
Bisoprolol	66722-44-9	Beta-blocker	Surface Water	2024	4,5	ng/L	[6]
Bisoprolol	66722-44-9	Beta-blocker	Surface Water	2024	1199	ng/L	[6]
Bisoprolol	66722-44-9	Beta-blocker	Surface Water	2024	2360	ng/L	[6]
Bisoprolol	66722-44-9	Beta-blocker	Surface Water	2009	15,4	ng/L	[10]
Bisoprolol	66722-44-9	Beta-blocker	Surface Water	2009	9,8	ng/L	[10]
Bisoprolol	66722-44-9	Beta-blocker	Surface Water	2009	11,9	ng/L	[10]
Bisoprolol	66722-44-9	Beta-blocker	Surface Water	2009	24	ng/L	[10]
Bisoprolol	66722-44-9	Beta-blocker	Surface Water	2009	11,9	ng/L	[10]
Bisoprolol	66722-44-9	Beta-blocker	Surface Water	2009	26	ng/L	[10]
Bisoprolol	66722-44-9	Beta-blocker	Surface Water	2009	9,49	ng/L	[10]
Bisoprolol	66722-44-9	Beta-blocker	Surface Water	2009	19,1	ng/L	[10]
Bupropion	34911-55-2	Antidepressant	Surface Water	2018	15,7	ng/L	[1]
Bupropion	34911-55-2	Antidepressant	Surface Water	2018	27,1	ng/L	[1]
Bupropion	34911-55-2	Antidepressant	Surface Water	2019	28,5	ng/L	[1]
Bupropion	34911-55-2	Antidepressant	Surface Water	2018	60,6	ng/L	[1]
Carbamazepine	298-46-4	Antiepileptic	Tap Water	2015	3,34	ng/L	[15]
Carbamazepine	298-46-4	Antiepileptic	Tap Water	2015	20	ng/L	[16]
Carbamazepine	298-46-4	Antiepileptic	Tap Water	2015	22,3	ng/L	[16]
Carbamazepine	298-46-4	Antiepileptic	Surface Water	2019	0,593	ng/L	[1]
Carbamazepine	298-46-4	Antiepileptic	Surface Water	2019	2,43	ng/L	[1]
Carbamazepine	298-46-4	Antiepileptic	Surface Water	2010	146	ng/L	[17]
Carbamazepine	298-46-4	Antiepileptic	Surface Water	2010	110	ng/L	[17]
Carbamazepine	298-46-4	Antiepileptic	Surface Water	2017	354	ng/L	[8]
Carbamazepine	298-46-4	Antiepileptic	Surface Water	2017	206	ng/L	[8]
Carbamazepine	298-46-4	Antiepileptic	Surface Water	2018	8,5	ng/L	[1]
Carbamazepine	298-46-4	Antiepileptic	Surface Water	2018	6,96	ng/L	[14]
Carbamazepine	298-46-4	Antiepileptic	Surface Water	2018	6,75	ng/L	[14]
Carbamazepine	298-46-4	Antiepileptic	Surface Water	2018	6,07	ng/L	[14]
Carbamazepine	298-46-4	Antiepileptic	Surface Water	2018	7,57	ng/L	[14]
Carbamazepine	298-46-4	Antiepileptic	Surface Water	2018	4,99	ng/L	[14]
Carbamazepine	298-46-4	Antiepileptic	Surface Water	2018	5,39	ng/L	[14]
Carbamazepine	298-46-4	Antiepileptic	Surface Water	2018	7,33	ng/L	[14]
Carbamazepine	298-46-4	Antiepileptic	Surface Water	2018	6,62	ng/L	[14]
Carbamazepine	298-46-4	Antiepileptic	Surface Water	2018	7,24	ng/L	[14]
Carbamazepine	298-46-4	Antiepileptic	Surface Water	2018	6,48	ng/L	[14]
Carbamazepine	298-46-4	Antiepileptic	Surface Water	2018	52,6	ng/L	[14]
Carbamazepine	298-46-4	Antiepileptic	Surface Water	2018	27,7	ng/L	[14]
Carbamazepine	298-46-4	Antiepileptic	Surface Water	2018	42,2	ng/L	[14]
Carbamazepine	298-46-4	Antiepileptic	Surface Water	2018	36,5	ng/L	[14]
Carbamazepine	298-46-4	Antiepileptic	Surface Water	2018	23,2	ng/L	[14]
Carbamazepine	298-46-4	Antiepileptic	Surface Water	2018	26,6	ng/L	[14]
Carbamazepine	298-46-4	Antiepileptic	Surface Water	2019	21,8	ng/L	[1]
Carbamazepine	298-46-4	Antiepileptic	Surface Water	2014	24,9	ng/L	[1]
Carbamazepine	298-46-4	Antiepileptic	Surface Water	2014	25,1	ng/L	[1]
Carbamazepine	298-46-4	Antiepileptic	Surface Water	2014	25,2	ng/L	[1]
Carbamazepine	298-46-4	Antiepileptic	Surface Water	2014	25,2	ng/L	[1]
Carbamazepine	298-46-4	Antiepileptic	Surface Water	2014	25,2	ng/L	[1]

Carbamazepine	298-46-4	Antiepileptic	Surface Water	2014	25,5	ng/L	[1]
Carbamazepine	298-46-4	Antiepileptic	Surface Water	2014	25,8	ng/L	[1]
Carbamazepine	298-46-4	Antiepileptic	Surface Water	2014	25,9	ng/L	[1]
Carbamazepine	298-46-4	Antiepileptic	Surface Water	2014	25,9	ng/L	[1]
Carbamazepine	298-46-4	Antiepileptic	Surface Water	2014	27,1	ng/L	[1]
Carbamazepine	298-46-4	Antiepileptic	Surface Water	2014	27,1	ng/L	[1]
Carbamazepine	298-46-4	Antiepileptic	Surface Water	2014	27,2	ng/L	[1]
Carbamazepine	298-46-4	Antiepileptic	Surface Water	2014	27,5	ng/L	[1]
Carbamazepine	298-46-4	Antiepileptic	Surface Water	2013	27,7	ng/L	[1]
Carbamazepine	298-46-4	Antiepileptic	Surface Water	2014	27,7	ng/L	[1]
Carbamazepine	298-46-4	Antiepileptic	Surface Water	2014	27,8	ng/L	[1]
Carbamazepine	298-46-4	Antiepileptic	Surface Water	2014	28,1	ng/L	[1]
Carbamazepine	298-46-4	Antiepileptic	Surface Water	2013	29	ng/L	[1]
Carbamazepine	298-46-4	Antiepileptic	Surface Water	2014	29,6	ng/L	[1]
Carbamazepine	298-46-4	Antiepileptic	Surface Water	2013	29,7	ng/L	[1]
Carbamazepine	298-46-4	Antiepileptic	Surface Water	2014	29,7	ng/L	[1]
Carbamazepine	298-46-4	Antiepileptic	Surface Water	2014	30,1	ng/L	[1]
Carbamazepine	298-46-4	Antiepileptic	Surface Water	2013	30,3	ng/L	[1]
Carbamazepine	298-46-4	Antiepileptic	Surface Water	2014	30,4	ng/L	[1]
Carbamazepine	298-46-4	Antiepileptic	Surface Water	2014	30,7	ng/L	[1]
Carbamazepine	298-46-4	Antiepileptic	Surface Water	2014	30,8	ng/L	[1]
Carbamazepine	298-46-4	Antiepileptic	Surface Water	2013	30,9	ng/L	[1]
Carbamazepine	298-46-4	Antiepileptic	Surface Water	2014	31,7	ng/L	[1]
Carbamazepine	298-46-4	Antiepileptic	Surface Water	2014	32,1	ng/L	[1]
Carbamazepine	298-46-4	Antiepileptic	Surface Water	2014	32,9	ng/L	[1]
Carbamazepine	298-46-4	Antiepileptic	Surface Water	2013	32,9	ng/L	[1]
Carbamazepine	298-46-4	Antiepileptic	Surface Water	2015	32,9	ng/L	[16]
Carbamazepine	298-46-4	Antiepileptic	Surface Water	2013	33,2	ng/L	[1]
Carbamazepine	298-46-4	Antiepileptic	Surface Water	2013	33,6	ng/L	[1]
Carbamazepine	298-46-4	Antiepileptic	Surface Water	2013	34,4	ng/L	[1]
Carbamazepine	298-46-4	Antiepileptic	Surface Water	2015	34,4	ng/L	[16]
Carbamazepine	298-46-4	Antiepileptic	Surface Water	2013	34,8	ng/L	[1]
Carbamazepine	298-46-4	Antiepileptic	Surface Water	2014	36,3	ng/L	[1]
Carbamazepine	298-46-4	Antiepileptic	Surface Water	2014	36,5	ng/L	[1]
Carbamazepine	298-46-4	Antiepileptic	Surface Water	2014	36,8	ng/L	[1]
Carbamazepine	298-46-4	Antiepileptic	Surface Water	2013	36,9	ng/L	[1]
Carbamazepine	298-46-4	Antiepileptic	Surface Water	2013	37,3	ng/L	[1]
Carbamazepine	298-46-4	Antiepileptic	Surface Water	2014	39,7	ng/L	[1]
Carbamazepine	298-46-4	Antiepileptic	Surface Water	2014	39,9	ng/L	[1]
Carbamazepine	298-46-4	Antiepileptic	Surface Water	2014	41,5	ng/L	[1]
Carbamazepine	298-46-4	Antiepileptic	Surface Water	2018	41,7	ng/L	[1]
Carbamazepine	298-46-4	Antiepileptic	Surface Water	2014	42,2	ng/L	[1]
Carbamazepine	298-46-4	Antiepileptic	Surface Water	2013	45,1	ng/L	[1]
Carbamazepine	298-46-4	Antiepileptic	Surface Water	2019	45,7	ng/L	[1]
Carbamazepine	298-46-4	Antiepileptic	Surface Water	2013	46,8	ng/L	[1]
Carbamazepine	298-46-4	Antiepileptic	Surface Water	2014	50,3	ng/L	[1]
Carbamazepine	298-46-4	Antiepileptic	Surface Water	2014	51,2	ng/L	[1]
Carbamazepine	298-46-4	Antiepileptic	Surface Water	2013	52,7	ng/L	[1]
Carbamazepine	298-46-4	Antiepileptic	Surface Water	2013	64,4	ng/L	[1]
Carbamazepine	298-46-4	Antiepileptic	Surface Water	2014	68,9	ng/L	[1]

Carbamazepine	298-46-4	Antiepileptic	Surface Water	2013	112,8	ng/L	[1]
Carbamazepine	298-46-4	Antiepileptic	Surface Water	2013	142,8	ng/L	[1]
Carbamazepine	298-46-4	Antiepileptic	Surface Water	2013	143	ng/L	[2]
Carbamazepine	298-46-4	Antiepileptic	Surface Water	2018	166	ng/L	[1]
Carbamazepine	298-46-4	Antiepileptic	Surface Water	2013	173	ng/L	[1]
Carbamazepine	298-46-4	Antiepileptic	Surface Water	2019	185	ng/L	[1]
Carbamazepine	298-46-4	Antiepileptic	Surface Water	2013	213,7	ng/L	[2]
Carbamazepine	298-46-4	Antiepileptic	Surface Water	2013	214	ng/L	[1]
Carbamazepine	298-46-4	Antiepileptic	Surface Water	2018	266	ng/L	[1]
Carbamazepine	298-46-4	Antiepileptic	Surface Water	2007	0,37	ng/L	[18]
Carbamazepine	298-46-4	Antiepileptic	Surface Water	2007	178	ng/L	[18]
Carbamazepine	298-46-4	Antiepileptic	Surface Water	2007	15,4	ng/L	[18]
Carbamazepine	298-46-4	Antiepileptic	Surface Water	2024	18	ng/L	[6]
Carbamazepine	298-46-4	Antiepileptic	Bottled water	2013	1,9	ng/L	[7]
Carbamazepine	298-46-4	Antiepileptic	Bottled water	2013	14	ng/L	[7]
Carbamazepine	298-46-4	Antiepileptic	Bottled water	2015	22,1	ng/L	[16]
Carboxyibuprofen	15935-54-3	Metabolic	Surface Water	2019	43,8	ng/L	[1]
Carboxyibuprofen	15935-54-3	Metabolic	Surface Water	2019	109	ng/L	[1]
Carboxyibuprofen	15935-54-3	Metabolic	Surface Water	2018	1227	ng/L	[1]
Cetirizine	83881-51-0	Antihistamine	Surface Water	2010	40	ng/L	[17]
Chlorfenvinphos	470-90-6	Antiparasitics	Tap Water	2015	6,5	ng/L	[15]
Chlorfenvinphos	470-90-6	Antiparasitics	Tap Water	2015	2,46	ng/L	[15]
Chlorfenvinphos	470-90-6	Antiparasitics	Bottled water	2015	3,89	ng/L	[15]
Chlorfenvinphos	470-90-6	Antiparasitics	Bottled water	2015	0,49	ng/L	[15]
Ciprofloxacin	85721-33-1	Antibiotics	Surface Water	2017	339	ng/L	[8]
Ciprofloxacin	85721-33-1	Antibiotics	Surface Water	2006	119,2	ng/L	[19]
Ciprofloxacin	85721-33-1	Antibiotics	Surface Water	2006	79,6	ng/L	[19]
Ciprofloxacin	85721-33-1	Antibiotics	Surface Water	2013	88,7	ng/L	[2]
Ciprofloxacin	85721-33-1	Antibiotics	Surface Water	2009	59,3	ng/L	[10]
Citalopram	59729-33-8	Antidepressant	Surface Water	2013	1,67	ng/L	[2]
Citalopram	59729-33-8	Antidepressant	Surface Water	2019	6,64	ng/L	[1]
Citalopram	59729-33-8	Antidepressant	Surface Water	2018	14,4	ng/L	[1]
Citalopram	59729-33-8	Antidepressant	Surface Water	2014	17,8	ng/L	[1]
Citalopram	59729-33-8	Antidepressant	Surface Water	2017	67,9	ng/L	[8]
Citalopram	59729-33-8	Antidepressant	Surface Water	2014	53	ng/L	[9]
Citalopram	59729-33-8	Antidepressant	Surface Water	2014	39,21	ng/L	[9]
Citalopram	59729-33-8	Antidepressant	Surface Water	2014	20,7	ng/L	[9]
Citalopram	59729-33-8	Antidepressant	Surface Water	2013	28,9	ng/L	[1]
Citalopram	59729-33-8	Antidepressant	Surface Water	2018	41,5	ng/L	[1]
Citalopram	59729-33-8	Antidepressant	Surface Water	2019	46,2	ng/L	[1]
Citalopram propionic acid	10403174	Metabolic	Surface Water	2018	9,3	ng/L	[1]
Citalopram propionic acid	10403174	Metabolic	Surface Water	2018	11,1	ng/L	[1]
Citalopram propionic acid	10403174	Metabolic	Surface Water	2018	19,4	ng/L	[1]
Citalopram propionic acid	10403174	Metabolic	Surface Water	2018	24	ng/L	[1]
Clarithromycin	81103-11-9	Antibiotics	Surface Water	2013	8,67	ng/L	[2]
Clarithromycin	81103-11-9	Antibiotics	Surface Water	2019	9,73	ng/L	[1]
Clarithromycin	81103-11-9	Antibiotics	Surface Water	2013	21,9	ng/L	[2]
Clarithromycin	81103-11-9	Antibiotics	Surface Water	2018	23	ng/L	[1]
Clarithromycin	81103-11-9	Antibiotics	Surface Water	2017	269	ng/L	[8]
Clarithromycin	81103-11-9	Antibiotics	Surface Water	2017	76,6	ng/L	[8]

Clarithromycin	81103-11-9	Antibiotics	Surface Water	2014	39,1	ng/L	[9]
Clarithromycin	81103-11-9	Antibiotics	Surface Water	2014	33,08	ng/L	[9]
Clarithromycin	81103-11-9	Antibiotics	Surface Water	2014	24,8	ng/L	[9]
Clarithromycin	81103-11-9	Antibiotics	Surface Water	2018	0,48	ng/L	[14]
Clarithromycin	81103-11-9	Antibiotics	Surface Water	2018	0,62	ng/L	[14]
Clarithromycin	81103-11-9	Antibiotics	Surface Water	2018	0,48	ng/L	[14]
Clarithromycin	81103-11-9	Antibiotics	Surface Water	2018	0,46	ng/L	[14]
Clarithromycin	81103-11-9	Antibiotics	Surface Water	2018	0,45	ng/L	[14]
Clarithromycin	81103-11-9	Antibiotics	Surface Water	2018	0,51	ng/L	[14]
Clarithromycin	81103-11-9	Antibiotics	Surface Water	2018	0,29	ng/L	[14]
Clarithromycin	81103-11-9	Antibiotics	Surface Water	2018	0,35	ng/L	[14]
Clarithromycin	81103-11-9	Antibiotics	Surface Water	2018	0,68	ng/L	[14]
Clarithromycin	81103-11-9	Antibiotics	Surface Water	2018	0,46	ng/L	[14]
Clarithromycin	81103-11-9	Antibiotics	Surface Water	2018	4,65	ng/L	[14]
Clarithromycin	81103-11-9	Antibiotics	Surface Water	2018	2,62	ng/L	[14]
Clarithromycin	81103-11-9	Antibiotics	Surface Water	2018	1,46	ng/L	[14]
Clarithromycin	81103-11-9	Antibiotics	Surface Water	2018	1,54	ng/L	[14]
Clarithromycin	81103-11-9	Antibiotics	Surface Water	2018	1,69	ng/L	[14]
Clarithromycin	81103-11-9	Antibiotics	Surface Water	2018	3,04	ng/L	[14]
Clarithromycin	81103-11-9	Antibiotics	Surface Water	2013	26,8	ng/L	[2]
Clarithromycin	81103-11-9	Antibiotics	Surface Water	2019	31,6	ng/L	[1]
Clarithromycin	81103-11-9	Antibiotics	Surface Water	2019	69,4	ng/L	[1]
Clarithromycin	81103-11-9	Antibiotics	Surface Water	2018	99,1	ng/L	[1]
Clarithromycin	81103-11-9	Antibiotics	Surface Water	2018	187	ng/L	[1]
Clarithromycin	81103-11-9	Antibiotics	Surface Water	2024	1,8	ng/L	[6]
Clofibric	882-09-7	Antihyperlipidemic	Surface Water	2024	8,9	ng/L	[6]
Clofibric	882-09-7	Antihyperlipidemic	Surface Water	2024	36,1	ng/L	[6]
Clofibric	882-09-7	Antihyperlipidemic	Surface Water	2024	1165	ng/L	[6]
Cocaine	50-36-2	Drug	Tap Water	2020	340	ng/L	[11]
Cocaine	50-36-2	Drug	Tap Water	2020	40	ng/L	[11]
Cocaine	50-36-2	Drug	Tap Water	2020	119	ng/L	[11]
Codeine	76-57-3	Analgesics	Surface Water	2024	1,5	ng/L	[6]
Codeine	76-57-3	Analgesics	Surface Water	2024	1,8	ng/L	[6]
Codeine	76-57-3	Analgesics	Surface Water	2024	2,1	ng/L	[6]
Diazepam	439-14-5	Antidepressant	Surface Water	2018	13,9	ng/L	[1]
Diazepam	439-14-5	Antidepressant	Surface Water	2007	3,65	ng/L	[18]
Diclofenac	15307-86-5	Analgesics	Surface Water	2018	13,1	ng/L	[1]
Diclofenac	15307-86-5	Analgesics	Surface Water	2014	51,2	ng/L	[9]
Diclofenac	15307-86-5	Analgesics	Surface Water	2014	33,56	ng/L	[9]
Diclofenac	15307-86-5	Analgesics	Surface Water	2014	25,13	ng/L	[9]
Diclofenac	15307-86-5	Analgesics	Surface Water	2018	3,08	ng/L	[14]
Diclofenac	15307-86-5	Analgesics	Surface Water	2018	2,35	ng/L	[14]
Diclofenac	15307-86-5	Analgesics	Surface Water	2018	2,04	ng/L	[14]
Diclofenac	15307-86-5	Analgesics	Surface Water	2018	2,91	ng/L	[14]
Diclofenac	15307-86-5	Analgesics	Surface Water	2018	1,82	ng/L	[14]
Diclofenac	15307-86-5	Analgesics	Surface Water	2018	1,35	ng/L	[14]
Diclofenac	15307-86-5	Analgesics	Surface Water	2018	2,06	ng/L	[14]
Diclofenac	15307-86-5	Analgesics	Surface Water	2018	2,05	ng/L	[14]
Diclofenac	15307-86-5	Analgesics	Surface Water	2018	1,19	ng/L	[14]
Diclofenac	15307-86-5	Analgesics	Surface Water	2018	0,99	ng/L	[14]

Diclofenac	15307-86-5	Analgesics	Surface Water	2018	59,8	ng/L	[14]
Diclofenac	15307-86-5	Analgesics	Surface Water	2018	11,7	ng/L	[14]
Diclofenac	15307-86-5	Analgesics	Surface Water	2018	17,6	ng/L	[14]
Diclofenac	15307-86-5	Analgesics	Surface Water	2018	13,7	ng/L	[14]
Diclofenac	15307-86-5	Analgesics	Surface Water	2018	5,11	ng/L	[14]
Diclofenac	15307-86-5	Analgesics	Surface Water	2018	14,4	ng/L	[14]
Diclofenac	15307-86-5	Analgesics	Surface Water	2013	38	ng/L	[2]
Diclofenac	15307-86-5	Analgesics	Surface Water	2019	72,5	ng/L	[1]
Diclofenac	15307-86-5	Analgesics	Surface Water	2018	112	ng/L	[1]
Diclofenac	15307-86-5	Analgesics	Surface Water	2019	119	ng/L	[1]
Diclofenac	15307-86-5	Analgesics	Surface Water	2018	285	ng/L	[1]
Diclofenac	15307-86-5	Analgesics	Surface Water	2019	848	ng/L	[1]
Diclofenac	15307-86-5	Analgesics	Surface Water	2024	11,8	ng/L	[6]
Diclofenac	15307-86-5	Analgesics	Surface Water	2024	27,3	ng/L	[6]
Diclofenac	15307-86-5	Analgesics	Surface Water	2024	3165	ng/L	[6]
Diclofenac	15307-86-5	Analgesics	Surface Water	2009	56,8	ng/L	[10]
Diclofenac	15307-86-5	Analgesics	Surface Water	2009	22,8	ng/L	[10]
Diclofenac	15307-86-5	Analgesics	Bottled water	2015	3,95	ng/L	[15]
Diclofenac	15307-86-5	Analgesics	Bottled water	2015	7,66	ng/L	[15]
Diclofenac	15307-86-5	Analgesics	Tap Water	2015	7,87	ng/L	[15]
Diltiazem	42399-41-7	Benzothiazepines	Surface Water	2019	25,6	ng/L	[1]
Enrofloxacin	93106-60-6	Antibiotics	Surface Water	2006	102,5	ng/L	[19]
Enrofloxacin	93106-60-6	Antibiotics	Surface Water	2006	67	ng/L	[19]
Erythromycin	114-07-8	Antibiotic	Bottled water	2013	0,5	ng/L	[7]
Erythromycin	114-07-8	Antibiotic	Bottled water	2013	5,69	ng/L	[7]
Erythromycin	114-07-8	Antibiotics	Surface Water	2014	38,8	ng/L	[9]
Erythromycin	114-07-8	Antibiotics	Surface Water	2014	35,51	ng/L	[9]
Erythromycin	114-07-8	Antibiotics	Surface Water	2014	32,89	ng/L	[9]
Erythromycin	114-07-8	Antibiotics	Surface Water	2018	0,06	ng/L	[14]
Erythromycin	114-07-8	Antibiotics	Surface Water	2018	3,76	ng/L	[14]
Erythromycin	114-07-8	Antibiotics	Surface Water	2018	1,51	ng/L	[14]
Erythromycin	114-07-8	Antibiotics	Surface Water	2018	0,42	ng/L	[14]
Erythromycin	114-07-8	Antibiotics	Surface Water	2018	0,31	ng/L	[14]
Erythromycin	114-07-8	Antibiotics	Surface Water	2018	0,26	ng/L	[14]
Erythromycin	114-07-8	Antibiotics	Surface Water	2018	0,21	ng/L	[14]
Estrone	53-16-7	Estrogen	Surface Water	2009	9,9	ng/L	[4]
Estrone	53-16-7	Estrogen	Surface Water	2009	7,6	ng/L	[4]
Estrone	53-16-7	Estrogen	Surface Water	2009	9,4	ng/L	[4]
Estrone	53-16-7	Estrogen	Surface Water	2009	8,5	ng/L	[4]
Estrone	53-16-7	Estrogen	Surface Water	2009	10,4	ng/L	[4]
Estrone	53-16-7	Estrogen	Surface Water	2009	5,8	ng/L	[4]
Estrone	53-16-7	Estrogen	Surface Water	2010	2,6	ng/L	[5]
Estrone	53-16-7	Estrogen	Surface Water	2010	3,7	ng/L	[5]
Estrone	53-16-7	Estrogen	Surface Water	2010	1,4	ng/L	[5]
Estrone	53-16-7	Estrogen	Surface Water	2010	4,1	ng/L	[5]
Fenofibrate acid	42017-89-0	Antilipemic	Surface Water	2007	1,48	ng/L	[18]
Fenofibrate acid	42017-89-0	Antilipemic	Surface Water	2007	70,3	ng/L	[18]
Fenofibrate acid	42017-89-0	Antilipemic	Surface Water	2007	16,8	ng/L	[18]
Fluconazole	86386-73-4	Antifungal	Surface Water	2016	266,4	ng/L	[13]
Fluconazole	86386-73-4	Antifungal	Surface Water	2016	355,9	ng/L	[13]

Fluconazole	86386-73-4	Antifungal	Surface Water	2016	573,8	ng/L	[13]
Fluconazole	86386-73-4	Antifungal	Surface Water	2016	227,5	ng/L	[13]
Fluoxetine	54910-89-3	Antidepressant	Tap Water	2015	0,27	ng/L	[16]
Fluoxetine	54910-89-3	Antidepressant	Tap Water	2015	1,9	ng/L	[16]
Fluoxetine	54910-89-3	Antidepressant	Surface Water	2014	2,01	ng/L	[1]
Fluoxetine	54910-89-3	Antidepressant	Surface Water	2014	2,07	ng/L	[1]
Fluoxetine	54910-89-3	Antidepressant	Surface Water	2014	2,15	ng/L	[1]
Fluoxetine	54910-89-3	Antidepressant	Surface Water	2013	2,17	ng/L	[1]
Fluoxetine	54910-89-3	Antidepressant	Surface Water	2014	2,18	ng/L	[1]
Fluoxetine	54910-89-3	Antidepressant	Surface Water	2013	2,25	ng/L	[1]
Fluoxetine	54910-89-3	Antidepressant	Surface Water	2014	2,26	ng/L	[1]
Fluoxetine	54910-89-3	Antidepressant	Surface Water	2014	2,29	ng/L	[1]
Fluoxetine	54910-89-3	Antidepressant	Surface Water	2014	2,34	ng/L	[1]
Fluoxetine	54910-89-3	Antidepressant	Surface Water	2013	2,38	ng/L	[1]
Fluoxetine	54910-89-3	Antidepressant	Surface Water	2013	2,44	ng/L	[1]
Fluoxetine	54910-89-3	Antidepressant	Surface Water	2013	2,46	ng/L	[1]
Fluoxetine	54910-89-3	Antidepressant	Surface Water	2013	2,48	ng/L	[1]
Fluoxetine	54910-89-3	Antidepressant	Surface Water	2014	2,52	ng/L	[1]
Fluoxetine	54910-89-3	Antidepressant	Surface Water	2014	2,53	ng/L	[1]
Fluoxetine	54910-89-3	Antidepressant	Surface Water	2013	2,57	ng/L	[1]
Fluoxetine	54910-89-3	Antidepressant	Surface Water	2013	2,6	ng/L	[1]
Fluoxetine	54910-89-3	Antidepressant	Surface Water	2014	2,67	ng/L	[1]
Fluoxetine	54910-89-3	Antidepressant	Surface Water	2014	2,72	ng/L	[1]
Fluoxetine	54910-89-3	Antidepressant	Surface Water	2014	2,83	ng/L	[1]
Fluoxetine	54910-89-3	Antidepressant	Surface Water	2014	2,87	ng/L	[1]
Fluoxetine	54910-89-3	Antidepressant	Surface Water	2014	2,89	ng/L	[1]
Fluoxetine	54910-89-3	Antidepressant	Surface Water	2013	2,92	ng/L	[1]
Fluoxetine	54910-89-3	Antidepressant	Surface Water	2014	3	ng/L	[1]
Fluoxetine	54910-89-3	Antidepressant	Surface Water	2013	3,04	ng/L	[1]
Fluoxetine	54910-89-3	Antidepressant	Surface Water	2014	3,08	ng/L	[1]
Fluoxetine	54910-89-3	Antidepressant	Surface Water	2013	3,11	ng/L	[1]
Fluoxetine	54910-89-3	Antidepressant	Surface Water	2014	3,25	ng/L	[1]
Fluoxetine	54910-89-3	Antidepressant	Surface Water	2015	3,3	ng/L	[16]
Fluoxetine	54910-89-3	Antidepressant	Surface Water	2013	3,32	ng/L	[1]
Fluoxetine	54910-89-3	Antidepressant	Surface Water	2014	3,45	ng/L	[1]
Fluoxetine	54910-89-3	Antidepressant	Surface Water	2014	3,57	ng/L	[1]
Fluoxetine	54910-89-3	Antidepressant	Surface Water	2014	3,63	ng/L	[1]
Fluoxetine	54910-89-3	Antidepressant	Surface Water	2014	3,69	ng/L	[1]
Fluoxetine	54910-89-3	Antidepressant	Surface Water	2015	3,7	ng/L	[16]
Fluoxetine	54910-89-3	Antidepressant	Surface Water	2013	3,74	ng/L	[1]
Fluoxetine	54910-89-3	Antidepressant	Surface Water	2014	3,82	ng/L	[1]
Fluoxetine	54910-89-3	Antidepressant	Surface Water	2014	3,85	ng/L	[1]
Fluoxetine	54910-89-3	Antidepressant	Surface Water	2013	4	ng/L	[1]
Fluoxetine	54910-89-3	Antidepressant	Surface Water	2014	4,04	ng/L	[1]
Fluoxetine	54910-89-3	Antidepressant	Surface Water	2014	4,19	ng/L	[1]
Fluoxetine	54910-89-3	Antidepressant	Surface Water	2014	5,34	ng/L	[1]
Fluoxetine	54910-89-3	Antidepressant	Surface Water	2014	5,58	ng/L	[1]
Fluoxetine	54910-89-3	Antidepressant	Surface Water	2014	5,63	ng/L	[1]
Fluoxetine	54910-89-3	Antidepressant	Surface Water	2013	5,76	ng/L	[1]
Fluoxetine	54910-89-3	Antidepressant	Surface Water	2019	5,79	ng/L	[1]

Fluoxetine	54910-89-3	Antidepressant	Surface Water	2013	5,81	ng/L	[1]
Fluoxetine	54910-89-3	Antidepressant	Surface Water	2014	6,04	ng/L	[1]
Fluoxetine	54910-89-3	Antidepressant	Surface Water	2014	6,1	ng/L	[1]
Fluoxetine	54910-89-3	Antidepressant	Surface Water	2019	6,42	ng/L	[1]
Fluoxetine	54910-89-3	Antidepressant	Surface Water	2014	6,52	ng/L	[1]
Fluoxetine	54910-89-3	Antidepressant	Surface Water	2013	7,1	ng/L	[1]
Fluoxetine	54910-89-3	Antidepressant	Surface Water	2019	7,19	ng/L	[1]
Fluoxetine	54910-89-3	Antidepressant	Surface Water	2014	7,64	ng/L	[1]
Fluoxetine	54910-89-3	Antidepressant	Surface Water	2014	8,2	ng/L	[1]
Fluoxetine	54910-89-3	Antidepressant	Surface Water	2013	8,25	ng/L	[1]
Fluoxetine	54910-89-3	Antidepressant	Surface Water	2019	8,53	ng/L	[1]
Fluoxetine	54910-89-3	Antidepressant	Surface Water	2014	10	ng/L	[1]
Fluoxetine	54910-89-3	Antidepressant	Surface Water	2013	15,9	ng/L	[1]
Fluoxetine	54910-89-3	Antidepressant	Surface Water	2013	17,6	ng/L	[1]
Fluoxetine	54910-89-3	Antidepressant	Surface Water	2014	19,5	ng/L	[1]
Fluoxetine	54910-89-3	Antidepressant	Surface Water	2019	21,1	ng/L	[1]
Fluoxetine	54910-89-3	Antidepressant	Surface Water	2017	2	ng/L	[8]
Fluoxetine	54910-89-3	Antidepressant	Surface Water	2017	1,9	ng/L	[8]
Fluoxetine	54910-89-3	Antidepressant	Surface Water	2017	1,9	ng/L	[8]
Fluoxetine	54910-89-3	Antidepressant	Surface Water	2017	28,9	ng/L	[8]
Fluoxetine	54910-89-3	Antidepressant	Surface Water	2017	6,7	ng/L	[8]
Fluoxetine	54910-89-3	Antidepressant	Surface Water	2014	25,37	ng/L	[9]
Fluoxetine	56296-78-7	Antidepressant	Surface Water	2018	14	ng/L	[12]
Furosemide	54-31-9	Diuretics	Surface Water	2024	66,7	ng/L	[6]
Furosemide	54-31-9	Diuretics	Surface Water	2024	3315	ng/L	[6]
Furosemide	54-31-9	Diuretics	Surface Water	2024	8216	ng/L	[6]
Furosemide	54-31-9	Diuretics	Surface Water	2009	119	ng/L	[10]
Furosemide	54-31-9	Diuretics	Surface Water	2009	69,2	ng/L	[10]
Furosemide	54-31-9	Diuretics	Surface Water	2009	114	ng/L	[10]
Furosemide	54-31-9	Diuretics	Surface Water	2009	192	ng/L	[10]
Furosemide	54-31-9	Diuretics	Surface Water	2009	110	ng/L	[10]
Furosemide	54-31-9	Diuretics	Surface Water	2009	283	ng/L	[10]
Furosemide	54-31-9	Diuretics	Surface Water	2009	227	ng/L	[10]
Gemfibrozil	25812-30-0	Lipid-lowering	Surface Water	2018	5,72	ng/L	[1]
Gemfibrozil	25812-30-0	Lipid-lowering	Surface Water	2018	9,46	ng/L	[1]
Gemfibrozil	25812-30-0	Lipid-lowering	Surface Water	2018	15,2	ng/L	[1]
Gemfibrozil	25812-30-0	Lipid-lowering	Surface Water	2019	25,8	ng/L	[1]
Gemfibrozil	25812-30-0	Lipid-lowering	Surface Water	2018	39,9	ng/L	[1]
Gemfibrozil	25812-30-0	Lipid-lowering	Surface Water	2014	10,3	ng/L	[9]
Gemfibrozil	25812-30-0	Lipid-lowering	Surface Water	2014	7,78	ng/L	[9]
Gemfibrozil	25812-30-0	Lipid-lowering	Surface Water	2014	6,69	ng/L	[9]
Gemfibrozil	25812-30-0	Lipid-lowering	Surface Water	2009	43,1	ng/L	[10]
Gemfibrozil	25812-30-0	Lipid-lowering	Surface Water	2009	35	ng/L	[10]
Gemfibrozil	25812-30-0	Lipid-lowering	Surface Water	2009	59,8	ng/L	[10]
Gemfibrozil	25812-30-0	Lipid-lowering	Surface Water	2009	43,3	ng/L	[10]
Gemfibrozil	25812-30-0	Lipid-lowering	Surface Water	2009	91,2	ng/L	[10]
Gemfibrozil	25812-30-0	Lipid-lowering	Surface Water	2009	74,2	ng/L	[10]
Hydrochlorothiazide	58-93-5	Diuretics	Surface Water	2009	31	ng/L	[20]
Hydrochlorothiazide	58-93-5	Diuretics	Surface Water	2009	57,8	ng/L	[10]
Hydrochlorothiazide	58-93-5	Diuretics	Surface Water	2009	389	ng/L	[10]

Hydrochlorothiazide	58-93-5	Diuretics	Surface Water	2009	352	ng/L	[10]
Hydrochlorothiazide	58-93-5	Diuretics	Surface Water	2009	86,7	ng/L	[10]
Hydrochlorothiazide	58-93-5	Diuretics	Surface Water	2009	196	ng/L	[10]
Hydroxyibuprofen	53949-53-4	Metabolic	Surface Water	2018	15,3	ng/L	[1]
Hydroxyibuprofen	53949-53-4	Metabolic	Surface Water	2013	16,3	ng/L	[2]
Hydroxyibuprofen	53949-53-4	Metabolic	Surface Water	2013	18,2	ng/L	[1]
Hydroxyibuprofen	53949-53-4	Metabolic	Surface Water	2014	18,9	ng/L	[1]
Hydroxyibuprofen	53949-53-4	Metabolic	Surface Water	2014	24,4	ng/L	[1]
Hydroxyibuprofen	53949-53-4	Metabolic	Surface Water	2013	28,6	ng/L	[1]
Hydroxyibuprofen	53949-53-4	Metabolic	Surface Water	2018	36,2	ng/L	[1]
Hydroxyibuprofen	53949-53-4	Metabolic	Surface Water	2013	40,3	ng/L	[2]
Hydroxyibuprofen	53949-53-4	Metabolic	Surface Water	2014	41	ng/L	[1]
Hydroxyibuprofen	53949-53-4	Metabolic	Surface Water	2019	49	ng/L	[1]
Hydroxyibuprofen	53949-53-4	Metabolic	Surface Water	2014	60,5	ng/L	[1]
Hydroxyibuprofen	53949-53-4	Metabolic	Surface Water	2019	65,4	ng/L	[1]
Hydroxyibuprofen	53949-53-4	Metabolic	Surface Water	2013	81,4	ng/L	[1]
Hydroxyibuprofen	53949-53-4	Metabolic	Surface Water	2014	87,4	ng/L	[1]
Hydroxyibuprofen	53949-53-4	Metabolic	Surface Water	2014	98,9	ng/L	[1]
Hydroxyibuprofen	53949-53-4	Metabolic	Surface Water	2013	142	ng/L	[1]
Hydroxyibuprofen	53949-53-4	Metabolic	Surface Water	2019	226	ng/L	[1]
Hydroxyibuprofen	53949-53-4	Metabolic	Surface Water	2019	300	ng/L	[1]
Hydroxyibuprofen	53949-53-4	Metabolic	Surface Water	2013	318	ng/L	[1]
Hydroxyibuprofen	53949-53-4	Metabolic	Surface Water	2018	388	ng/L	[1]
Hydroxyibuprofen	53949-53-4	Metabolic	Surface Water	2019	480	ng/L	[1]
Hydroxyibuprofen	53949-53-4	Metabolic	Surface Water	2018	1295	ng/L	[1]
Hydroxyibuprofen	53949-53-4	Metabolic	Surface Water	2018	1673	ng/L	[1]
Ibuprofen	15687-27-1	Analgesics	Surface Water	2018	1,38	ng/L	[1]
Ibuprofen	15687-27-1	Analgesics	Surface Water	2019	3,41	ng/L	[1]
Ibuprofen	15687-27-1	Analgesics	Surface Water	2018	4,78	ng/L	[1]
Ibuprofen	15687-27-1	Analgesics	Surface Water	2019	8,88	ng/L	[1]
Ibuprofen	15687-27-1	Analgesics	Surface Water	2013	35,2	ng/L	[1]
Ibuprofen	15687-27-1	Analgesics	Surface Water	2019	35,9	ng/L	[1]
Ibuprofen	15687-27-1	Analgesics	Surface Water	2010	40	ng/L	[21]
Ibuprofen	15687-27-1	Analgesics	Surface Water	2019	43,1	ng/L	[1]
Ibuprofen	15687-27-1	Analgesics	Surface Water	2018	45,5	ng/L	[1]
Ibuprofen	15687-27-1	Analgesics	Surface Water	2014	53,7	ng/L	[1]
Ibuprofen	15687-27-1	Analgesics	Surface Water	2010	55	ng/L	[21]
Ibuprofen	15687-27-1	Analgesics	Surface Water	2019	57,8	ng/L	[1]
Ibuprofen	15687-27-1	Analgesics	Surface Water	2013	62,3	ng/L	[1]
Ibuprofen	15687-27-1	Analgesics	Surface Water	2018	69,1	ng/L	[1]
Ibuprofen	15687-27-1	Analgesics	Surface Water	2014	77,9	ng/L	[1]
Ibuprofen	15687-27-1	Analgesics	Surface Water	2010	81	ng/L	[21]
Ibuprofen	15687-27-1	Analgesics	Surface Water	2013	81,5	ng/L	[1]
Ibuprofen	15687-27-1	Analgesics	Surface Water	2014	83,1	ng/L	[1]
Ibuprofen	15687-27-1	Analgesics	Surface Water	2018	94,7	ng/L	[1]
Ibuprofen	15687-27-1	Analgesics	Surface Water	2014	124	ng/L	[1]
Ibuprofen	15687-27-1	Analgesics	Surface Water	2014	129	ng/L	[1]
Ibuprofen	15687-27-1	Analgesics	Surface Water	2014	129	ng/L	[1]
Ibuprofen	15687-27-1	Analgesics	Surface Water	2010	138	ng/L	[21]
Ibuprofen	15687-27-1	Analgesics	Surface Water	2014	139	ng/L	[1]

Ibuprofen	15687-27-1	Analgesics	Surface Water	2014	144	ng/L	[1]
Ibuprofen	15687-27-1	Analgesics	Surface Water	2013	156	ng/L	[1]
Ibuprofen	15687-27-1	Analgesics	Surface Water	2014	169	ng/L	[1]
Ibuprofen	15687-27-1	Analgesics	Surface Water	2010	173	ng/L	[21]
Ibuprofen	15687-27-1	Analgesics	Surface Water	2010	185	ng/L	[21]
Ibuprofen	15687-27-1	Analgesics	Surface Water	2013	196	ng/L	[1]
Ibuprofen	15687-27-1	Analgesics	Surface Water	2010	201	ng/L	[21]
Ibuprofen	15687-27-1	Analgesics	Surface Water	2010	204	ng/L	[21]
Ibuprofen	15687-27-1	Analgesics	Surface Water	2010	229	ng/L	[21]
Ibuprofen	15687-27-1	Analgesics	Surface Water	2010	232	ng/L	[21]
Ibuprofen	15687-27-1	Analgesics	Surface Water	2014	238	ng/L	[1]
Ibuprofen	15687-27-1	Analgesics	Surface Water	2010	256	ng/L	[21]
Ibuprofen	15687-27-1	Analgesics	Surface Water	2014	277	ng/L	[1]
Ibuprofen	15687-27-1	Analgesics	Surface Water	2010	343	ng/L	[21]
Ibuprofen	15687-27-1	Analgesics	Surface Water	2014	352	ng/L	[1]
Ibuprofen	15687-27-1	Analgesics	Surface Water	2010	359	ng/L	[21]
Ibuprofen	15687-27-1	Analgesics	Surface Water	2013	360	ng/L	[1]
Ibuprofen	15687-27-1	Analgesics	Surface Water	2014	416	ng/L	[1]
Ibuprofen	15687-27-1	Analgesics	Surface Water	2014	426	ng/L	[1]
Ibuprofen	15687-27-1	Analgesics	Surface Water	2014	476	ng/L	[1]
Ibuprofen	15687-27-1	Analgesics	Surface Water	2014	491	ng/L	[1]
Ibuprofen	15687-27-1	Analgesics	Surface Water	2013	541	ng/L	[1]
Ibuprofen	15687-27-1	Analgesics	Surface Water	2013	635	ng/L	[1]
Ibuprofen	15687-27-1	Analgesics	Surface Water	2010	723	ng/L	[21]
Ibuprofen	15687-27-1	Analgesics	Surface Water	2014	801	ng/L	[1]
Ibuprofen	15687-27-1	Analgesics	Surface Water	2014	827	ng/L	[1]
Ibuprofen	15687-27-1	Analgesics	Surface Water	2014	875	ng/L	[1]
Ibuprofen	15687-27-1	Analgesics	Surface Water	2014	985	ng/L	[1]
Ibuprofen	15687-27-1	Analgesics	Surface Water	2014	1317	ng/L	[1]
Ibuprofen	15687-27-1	Analgesics	Surface Water	2024	14,7	ng/L	[6]
Ibuprofen	15687-27-1	Analgesics	Surface Water	2024	56,9	ng/L	[6]
Ibuprofen	15687-27-1	Analgesics	Surface Water	2024	3774	ng/L	[6]
Iohexol	66108-95-0	Contrast agent	Surface Water	2018	70,5	ng/L	[14]
Iohexol	66108-95-0	Contrast agent	Surface Water	2018	10,1	ng/L	[14]
Iomeprol	78649-41-9	Contrast agent	Surface Water	2018	7,19	ng/L	[14]
Iomeprol	78649-41-9	Contrast agent	Surface Water	2018	7,99	ng/L	[14]
Iomeprol	78649-41-9	Contrast agent	Surface Water	2018	8,17	ng/L	[14]
Iomeprol	78649-41-9	Contrast agent	Surface Water	2018	8,97	ng/L	[14]
Iomeprol	78649-41-9	Contrast agent	Surface Water	2018	5,5	ng/L	[14]
Iomeprol	78649-41-9	Contrast agent	Surface Water	2018	5,24	ng/L	[14]
Iomeprol	78649-41-9	Contrast agent	Surface Water	2018	6,67	ng/L	[14]
Iomeprol	78649-41-9	Contrast agent	Surface Water	2018	7,04	ng/L	[14]
Iomeprol	78649-41-9	Contrast agent	Surface Water	2018	6,56	ng/L	[14]
Iomeprol	78649-41-9	Contrast agent	Surface Water	2018	5,97	ng/L	[14]
Iomeprol	78649-41-9	Contrast agent	Surface Water	2018	386	ng/L	[14]
Iomeprol	78649-41-9	Contrast agent	Surface Water	2018	104	ng/L	[14]
Iomeprol	78649-41-9	Contrast agent	Surface Water	2018	156	ng/L	[14]
Iomeprol	78649-41-9	Contrast agent	Surface Water	2018	149	ng/L	[14]
Iomeprol	78649-41-9	Contrast agent	Surface Water	2018	129	ng/L	[14]
Iomeprol	78649-41-9	Contrast agent	Surface Water	2018	38,8	ng/L	[14]

Iopamidol	60166-93-0	Contrast agent	Surface Water	2018	4,31	ng/L	[14]
Iopromide	60166-93-0	Contrast agent	Surface Water	2018	70	ng/L	[14]
Iopromide	60166-93-0	Contrast agent	Surface Water	2018	61	ng/L	[14]
Iopromide	60166-93-0	Contrast agent	Surface Water	2018	70,7	ng/L	[14]
Iopromide	60166-93-0	Contrast agent	Surface Water	2018	70,9	ng/L	[14]
Iopromide	60166-93-0	Contrast agent	Surface Water	2018	43,1	ng/L	[14]
Iopromide	60166-93-0	Contrast agent	Surface Water	2018	50,2	ng/L	[14]
Iopromide	60166-93-0	Contrast agent	Surface Water	2018	64,2	ng/L	[14]
Iopromide	60166-93-0	Contrast agent	Surface Water	2018	67,3	ng/L	[14]
Iopromide	60166-93-0	Contrast agent	Surface Water	2018	67,6	ng/L	[14]
Iopromide	60166-93-0	Contrast agent	Surface Water	2018	50,2	ng/L	[14]
Iopromide	60166-93-0	Contrast agent	Surface Water	2018	2810	ng/L	[14]
Iopromide	60166-93-0	Contrast agent	Surface Water	2018	1230	ng/L	[14]
Iopromide	60166-93-0	Contrast agent	Surface Water	2018	1010	ng/L	[14]
Iopromide	60166-93-0	Contrast agent	Surface Water	2018	895	ng/L	[14]
Iopromide	60166-93-0	Contrast agent	Surface Water	2018	601	ng/L	[14]
Iopromide	60166-93-0	Contrast agent	Surface Water	2018	218	ng/L	[14]
Isoniazid	54-85-3	Antibiotics	Surface Water	2024	3,3	ng/L	[6]
Isoniazid	54-85-3	Antibiotics	Surface Water	2024	5,9	ng/L	[6]
Isoniazid	54-85-3	Antibiotics	Surface Water	2024	8,4	ng/L	[6]
Ketoprofen	22071-15-4	Analgesics	Surface Water	2013	8,22	ng/L	[1]
Ketoprofen	22071-15-4	Analgesics	Surface Water	2018	10,1	ng/L	[1]
Ketoprofen	22071-15-4	Analgesics	Surface Water	2014	15,4	ng/L	[1]
Ketoprofen	22071-15-4	Analgesics	Surface Water	2019	17,5	ng/L	[1]
Ketoprofen	22071-15-4	Analgesics	Surface Water	2019	18,8	ng/L	[1]
Ketoprofen	22071-15-4	Analgesics	Surface Water	2014	19,6	ng/L	[1]
Ketoprofen	22071-15-4	Analgesics	Surface Water	2014	19,6	ng/L	[1]
Ketoprofen	22071-15-4	Analgesics	Surface Water	2013	25,4	ng/L	[2]
Ketoprofen	22071-15-4	Analgesics	Surface Water	2019	28,6	ng/L	[1]
Ketoprofen	22071-15-4	Analgesics	Surface Water	2014	33,3	ng/L	[1]
Ketoprofen	22071-15-4	Analgesics	Surface Water	2013	41,6	ng/L	[1]
Ketoprofen	22071-15-4	Analgesics	Surface Water	2018	42,4	ng/L	[1]
Ketoprofen	22071-15-4	Analgesics	Surface Water	2013	52,1	ng/L	[1]
Ketoprofen	22071-15-4	Analgesics	Surface Water	2019	61,3	ng/L	[1]
Ketoprofen	22071-15-4	Analgesics	Surface Water	2019	71,7	ng/L	[1]
Ketoprofen	22071-15-4	Analgesics	Surface Water	2013	72,2	ng/L	[1]
Ketoprofen	22071-15-4	Analgesics	Surface Water	2013	75,3	ng/L	[2]
Ketoprofen	22071-15-4	Analgesics	Surface Water	2009	11	ng/L	[20]
Ketoprofen	22071-15-4	Analgesics	Surface Water	2024	17	ng/L	[6]
Ketoprofen	22071-15-4	Analgesics	Surface Water	2024	40,3	ng/L	[6]
Ketoprofen	22071-15-4	Analgesics	Surface Water	2024	63,5	ng/L	[6]
Ketoprofen	22071-15-4	Analgesics	Surface Water	2009	7,9	ng/L	[10]
Ketoprofen	22071-15-4	Analgesics	Surface Water	2009	11,9	ng/L	[10]
Lincomycin	154-21-2	Antibiotics	Surface Water	2018	0,25	ng/L	[14]
Lincomycin	154-21-2	Antibiotics	Surface Water	2018	0,24	ng/L	[14]
Lincomycin	154-21-2	Antibiotics	Surface Water	2018	0,25	ng/L	[14]
Lincomycin	154-21-2	Antibiotics	Surface Water	2018	0,27	ng/L	[14]
Lincomycin	154-21-2	Antibiotics	Surface Water	2018	0,23	ng/L	[14]
Lincomycin	154-21-2	Antibiotics	Surface Water	2018	0,25	ng/L	[14]
Lincomycin	154-21-2	Antibiotics	Surface Water	2018	0,26	ng/L	[14]

Lincomycin	154-21-2	Antibiotics	Surface Water	2018	0,28	ng/L	[14]
Lincomycin	154-21-2	Antibiotics	Surface Water	2018	0,32	ng/L	[14]
Lincomycin	154-21-2	Antibiotics	Surface Water	2018	0,26	ng/L	[14]
Lincomycin	154-21-2	Antibiotics	Surface Water	2018	0,7	ng/L	[14]
Lincomycin	154-21-2	Antibiotics	Surface Water	2018	0,54	ng/L	[14]
Lincomycin	154-21-2	Antibiotics	Surface Water	2018	0,6	ng/L	[14]
Lincomycin	154-21-2	Antibiotics	Surface Water	2018	0,59	ng/L	[14]
Lincomycin	154-21-2	Antibiotics	Surface Water	2018	0,46	ng/L	[14]
Lincomycin	154-21-2	Antibiotics	Surface Water	2018	0,57	ng/L	[14]
Lorazepam	846-49-1	Benzodiazepines	Surface Water	2009	49,1	ng/L	[10]
Lorazepam	846-49-1	Benzodiazepines	Surface Water	2009	41,3	ng/L	[10]
Lorazepam	846-49-1	Benzodiazepines	Surface Water	2009	21,1	ng/L	[10]
Lorazepam	846-49-1	Benzodiazepines	Surface Water	2009	25,8	ng/L	[10]
Metformin	657-24-9	Biguanides	Surface Water	2016	35,99	ng/L	[13]
Metformin	657-24-9	Biguanides	Surface Water	2016	35,99	ng/L	[13]
Metformin	657-24-9	Biguanides	Surface Water	2016	35,99	ng/L	[13]
Naproxen	22204-53-1	Analgesics	Surface Water	2018	12,3	ng/L	[1]
Naproxen	22204-53-1	Analgesics	Surface Water	2018	28,3	ng/L	[1]
Naproxen	22204-53-1	Analgesics	Surface Water	2019	40,9	ng/L	[1]
Naproxen	22204-53-1	Analgesics	Surface Water	2019	50,5	ng/L	[1]
Naproxen	22204-53-1	Analgesics	Surface Water	2014	55,5	ng/L	[1]
Naproxen	22204-53-1	Analgesics	Surface Water	2014	57	ng/L	[1]
Naproxen	22204-53-1	Analgesics	Surface Water	2014	72,7	ng/L	[1]
Naproxen	22204-53-1	Analgesics	Surface Water	2019	130	ng/L	[1]
Naproxen	22204-53-1	Analgesics	Surface Water	2018	156	ng/L	[1]
Naproxen	22204-53-1	Analgesics	Surface Water	2013	176	ng/L	[1]
Naproxen	22204-53-1	Analgesics	Surface Water	2013	202	ng/L	[1]
Naproxen	22204-53-1	Analgesics	Surface Water	2013	260	ng/L	[2]
Naproxen	22204-53-1	Analgesics	Surface Water	2024	1266	ng/L	[6]
Naproxen	22204-53-1	Analgesics	Surface Water	2009	86	ng/L	[10]
Naproxen	22204-53-1	Analgesics	Surface Water	2009	136	ng/L	[10]
Naproxen	22204-53-1	Analgesics	Surface Water	2009	91,3	ng/L	[10]
Nimesulide	51803-78-2	Anti-inflammatory	Surface Water	2019	6,5	ng/L	[1]
Ofloxacin	82419-36-1	Antibiotics	Surface Water	2017	120	ng/L	[8]
Omeprazole	73590-58-6	Proton-pump inhibitors	Surface Water	2024	11,1	ng/L	[6]
Omeprazole	73590-58-6	Proton-pump inhibitors	Surface Water	2024	1403	ng/L	[6]
Omeprazole	73590-58-6	Proton-pump inhibitors	Surface Water	2024	8255	ng/L	[6]
p-Aminophenol	123-30-8	Analgesics	Surface Water	2011	1630	ng/L	[22]
p-Aminophenol	123-30-8	Analgesics	Surface Water	2011	1250	ng/L	[22]
p-Aminophenol	123-30-8	Analgesics	Surface Water	2011	400	ng/L	[22]
p-Aminophenol	123-30-8	Analgesics	Surface Water	2011	520	ng/L	[22]
Paracetamol	103-90-2	Analgesics	Surface Water	2015	4,9	ng/L	[16]
Paracetamol	103-90-2	Analgesics	Surface Water	2013	47	ng/L	[2]
Paracetamol	103-90-2	Analgesics	Surface Water	2013	59,2	ng/L	[2]
Paracetamol	103-90-2	Analgesics	Surface Water	2014	69,2	ng/L	[9]
Paracetamol	103-90-2	Analgesics	Surface Water	2014	38,18	ng/L	[9]
Paracetamol	103-90-2	Analgesics	Surface Water	2011	170	ng/L	[22]
Paracetamol	103-90-2	Analgesics	Surface Water	2011	250	ng/L	[22]
Paracetamol	103-90-2	Analgesics	Surface Water	2009	63	ng/L	[20]
Paracetamol	103-90-2	Analgesics	Surface Water	2018	0,17	ng/L	[14]

Paracetamol	103-90-2	Analgesics	Surface Water	2018	0,28	ng/L	[14]
Paracetamol	103-90-2	Analgesics	Surface Water	2018	0,18	ng/L	[14]
Paracetamol	103-90-2	Analgesics	Surface Water	2018	0,16	ng/L	[14]
Paracetamol	103-90-2	Analgesics	Surface Water	2018	0,1	ng/L	[14]
Paracetamol	103-90-2	Analgesics	Surface Water	2018	0,21	ng/L	[14]
Paracetamol	103-90-2	Analgesics	Surface Water	2018	0,48	ng/L	[14]
Paracetamol	103-90-2	Analgesics	Surface Water	2018	0,27	ng/L	[14]
Paracetamol	103-90-2	Analgesics	Surface Water	2018	4,66	ng/L	[14]
Paracetamol	103-90-2	Analgesics	Surface Water	2018	2,79	ng/L	[14]
Paracetamol	103-90-2	Analgesics	Surface Water	2018	1,88	ng/L	[14]
Paracetamol	103-90-2	Analgesics	Surface Water	2018	2,61	ng/L	[14]
Paracetamol	103-90-2	Analgesics	Surface Water	2018	1,81	ng/L	[14]
Paracetamol	103-90-2	Analgesics	Surface Water	2018	2,35	ng/L	[14]
Paracetamol	103-90-2	Analgesics	Surface Water	2013	133	ng/L	[2]
Paracetamol	103-90-2	Analgesics	Surface Water	2013	327	ng/L	[2]
Paracetamol	103-90-2	Analgesics	Surface Water	2013	527	ng/L	[2]
Paracetamol	103-90-2	Analgesics	Surface Water	2009	37,6	ng/L	[10]
Paracetamol	103-90-2	Analgesics	Surface Water	2009	173	ng/L	[10]
Paracetamol	103-90-2	Analgesics	Surface Water	2009	368	ng/L	[10]
Paracetamol	103-90-2	Analgesics	Surface Water	2009	925	ng/L	[10]
Paracetamol	103-90-2	Analgesics	Surface Water	2009	491	ng/L	[10]
Paracetamol	103-90-2	Analgesics	Surface Water	2009	22,5	ng/L	[10]
Paracetamol-glucuronide	16110-10-4	Analgesics	Surface Water	2011	360	ng/L	[22]
Paracetamol-glucuronide	16110-10-4	Analgesics	Surface Water	2011	3570	ng/L	[22]
Paracetamol-glucuronide	16110-10-4	Analgesics	Surface Water	2011	180	ng/L	[22]
Paroxetine	61869-08-7	Antidepressant	Surface Water	2013	25,5	ng/L	[2]
Paroxetine	61869-08-7	Antidepressant	Surface Water	2013	25,5	ng/L	[2]
Paroxetine	61869-08-7	Antidepressant	Surface Water	2013	25,6	ng/L	[2]
Phenylbutazone	50-33-9	Anti-inflammatory	Surface Water	2016	132,7	ng/L	[13]
Phenylbutazone	50-33-9	Anti-inflammatory	Surface Water	2016	132,7	ng/L	[13]
Phenylbutazone	50-33-9	Anti-inflammatory	Surface Water	2016	132,7	ng/L	[13]
Prednisone	53-03-2	Glucocorticoids	Surface Water	2016	36,8	ng/L	[13]
Prednisone	53-03-2	Glucocorticoids	Surface Water	2016	36,8	ng/L	[13]
Prednisone	53-03-2	Glucocorticoids	Surface Water	2016	50,2	ng/L	[13]
Primidone	125-33-7	Anticonvulsant	Surface Water	2018	1,64	ng/L	[14]
Primidone	125-33-7	Anticonvulsant	Surface Water	2018	1,51	ng/L	[14]
Primidone	125-33-7	Anticonvulsant	Surface Water	2018	1,48	ng/L	[14]
Primidone	125-33-7	Anticonvulsant	Surface Water	2018	1,55	ng/L	[14]
Primidone	125-33-7	Anticonvulsant	Surface Water	2018	1,14	ng/L	[14]
Primidone	125-33-7	Anticonvulsant	Surface Water	2018	1,14	ng/L	[14]
Primidone	125-33-7	Anticonvulsant	Surface Water	2018	1,6	ng/L	[14]
Primidone	125-33-7	Anticonvulsant	Surface Water	2018	1,54	ng/L	[14]
Primidone	125-33-7	Anticonvulsant	Surface Water	2018	1,47	ng/L	[14]
Primidone	125-33-7	Anticonvulsant	Surface Water	2018	1,68	ng/L	[14]
Primidone	125-33-7	Anticonvulsant	Surface Water	2018	13,5	ng/L	[14]
Primidone	125-33-7	Anticonvulsant	Surface Water	2018	7,11	ng/L	[14]
Primidone	125-33-7	Anticonvulsant	Surface Water	2018	8,16	ng/L	[14]
Primidone	125-33-7	Anticonvulsant	Surface Water	2018	7,27	ng/L	[14]
Primidone	125-33-7	Anticonvulsant	Surface Water	2018	4,78	ng/L	[14]
Primidone	125-33-7	Anticonvulsant	Surface Water	2018	6,51	ng/L	[14]

Propranolol	525-66-6	beta blockers	Surface Water	2018	0,09	ng/L	[14]
Propranolol	525-66-6	beta blockers	Surface Water	2018	0,08	ng/L	[14]
Propranolol	525-66-6	beta blockers	Surface Water	2018	0,06	ng/L	[14]
Propranolol	525-66-6	beta blockers	Surface Water	2018	0,07	ng/L	[14]
Propranolol	525-66-6	beta blockers	Surface Water	2018	0,07	ng/L	[14]
Propranolol	525-66-6	beta blockers	Surface Water	2018	0,06	ng/L	[14]
Propranolol	525-66-6	beta blockers	Surface Water	2018	0,05	ng/L	[14]
Propranolol	525-66-6	beta blockers	Surface Water	2018	0,05	ng/L	[14]
Propranolol	525-66-6	beta blockers	Surface Water	2018	0,03	ng/L	[14]
Propranolol	525-66-6	beta blockers	Surface Water	2018	1,47	ng/L	[14]
Propranolol	525-66-6	beta blockers	Surface Water	2018	0,47	ng/L	[14]
Propranolol	525-66-6	beta blockers	Surface Water	2018	0,48	ng/L	[14]
Propranolol	525-66-6	beta blockers	Surface Water	2018	0,37	ng/L	[14]
Propranolol	525-66-6	beta blockers	Surface Water	2018	0,25	ng/L	[14]
Propranolol	525-66-6	beta blockers	Surface Water	2018	1,88	ng/L	[14]
Propranolol	525-66-6	beta blockers	Surface Water	2007	3,18	ng/L	[18]
Propranolol	525-66-6	beta blockers	Surface Water	2007	1,33	ng/L	[18]
Propranolol	525-66-6	beta blockers	Surface Water	2024	4,5	ng/L	[6]
Propranolol	525-66-6	beta blockers	Surface Water	2024	34,6	ng/L	[6]
Propranolol	525-66-6	beta blockers	Surface Water	2024	1159	ng/L	[6]
Ramipril	87333-19-5	Antihipertensive	Surface Water	2024	2,7	ng/L	[6]
Ramipril	87333-19-5	Antihipertensive	Surface Water	2024	77,5	ng/L	[6]
Ramipril	87333-19-5	Antihipertensive	Surface Water	2024	2659	ng/L	[6]
Roxithromycin	80214-83-1	Antibiotics	Surface Water	2018	0,12	ng/L	[14]
Roxithromycin	80214-83-1	Antibiotics	Surface Water	2018	0,08	ng/L	[14]
Roxithromycin	80214-83-1	Antibiotics	Surface Water	2018	0,2	ng/L	[14]
Salicylic acid	69-72-7	Analgesics	Tap Water	2015	39,4	ng/L	[16]
Salicylic acid	69-72-7	Analgesics	Bottled water	2015	21,2	ng/L	[16]
Salicylic acid	69-72-7	Analgesics	Bottled water	2015	30,6	ng/L	[16]
Salicylic acid	69-72-7	Analgesics	Tap Water	2015	66	ng/L	[16]
Salicylic acid	69-72-7	Analgesics	Surface Water	2014	25	ng/L	[1]
Salicylic acid	69-72-7	Analgesics	Surface Water	2014	32,8	ng/L	[1]
Salicylic acid	69-72-7	Analgesics	Surface Water	2014	35,6	ng/L	[1]
Salicylic acid	69-72-7	Analgesics	Surface Water	2014	36,8	ng/L	[1]
Salicylic acid	69-72-7	Analgesics	Surface Water	2014	40,9	ng/L	[1]
Salicylic acid	69-72-7	Analgesics	Surface Water	2013	43,1	ng/L	[1]
Salicylic acid	69-72-7	Analgesics	Surface Water	2014	43,9	ng/L	[1]
Salicylic acid	69-72-7	Analgesics	Surface Water	2014	46,1	ng/L	[1]
Salicylic acid	69-72-7	Analgesics	Surface Water	2014	46,6	ng/L	[1]
Salicylic acid	69-72-7	Analgesics	Surface Water	2014	49,3	ng/L	[1]
Salicylic acid	69-72-7	Analgesics	Surface Water	2014	49,6	ng/L	[1]
Salicylic acid	69-72-7	Analgesics	Surface Water	2014	52	ng/L	[1]
Salicylic acid	69-72-7	Analgesics	Surface Water	2014	53,6	ng/L	[1]
Salicylic acid	69-72-7	Analgesics	Surface Water	2014	53,8	ng/L	[1]
Salicylic acid	69-72-7	Analgesics	Surface Water	2014	61,3	ng/L	[1]
Salicylic acid	69-72-7	Analgesics	Surface Water	2014	64,8	ng/L	[1]
Salicylic acid	69-72-7	Analgesics	Surface Water	2014	68,8	ng/L	[1]
Salicylic acid	69-72-7	Analgesics	Surface Water	2014	69,1	ng/L	[1]
Salicylic acid	69-72-7	Analgesics	Surface Water	2013	72,2	ng/L	[1]
Salicylic acid	69-72-7	Analgesics	Surface Water	2014	77,6	ng/L	[1]

Salicylic acid	69-72-7	Analgesics	Surface Water	2013	78,5	ng/L	[1]
Salicylic acid	69-72-7	Analgesics	Surface Water	2014	81,2	ng/L	[1]
Salicylic acid	69-72-7	Analgesics	Surface Water	2014	85,1	ng/L	[1]
Salicylic acid	69-72-7	Analgesics	Surface Water	2014	86,1	ng/L	[1]
Salicylic acid	69-72-7	Analgesics	Surface Water	2014	87,2	ng/L	[1]
Salicylic acid	69-72-7	Analgesics	Surface Water	2013	89,1	ng/L	[1]
Salicylic acid	69-72-7	Analgesics	Surface Water	2013	89,2	ng/L	[1]
Salicylic acid	69-72-7	Analgesics	Surface Water	2015	89,2	ng/L	[16]
Salicylic acid	69-72-7	Analgesics	Surface Water	2014	90	ng/L	[1]
Salicylic acid	69-72-7	Analgesics	Surface Water	2014	91,5	ng/L	[1]
Salicylic acid	69-72-7	Analgesics	Surface Water	2013	91,9	ng/L	[1]
Salicylic acid	69-72-7	Analgesics	Surface Water	2013	95,1	ng/L	[1]
Salicylic acid	69-72-7	Analgesics	Surface Water	2013	95,4	ng/L	[1]
Salicylic acid	69-72-7	Analgesics	Surface Water	2014	97,6	ng/L	[1]
Salicylic acid	69-72-7	Analgesics	Surface Water	2014	99,2	ng/L	[1]
Salicylic acid	69-72-7	Analgesics	Surface Water	2014	100	ng/L	[1]
Salicylic acid	69-72-7	Analgesics	Surface Water	2014	107	ng/L	[1]
Salicylic acid	69-72-7	Analgesics	Surface Water	2013	110	ng/L	[1]
Salicylic acid	69-72-7	Analgesics	Surface Water	2013	114	ng/L	[1]
Salicylic acid	69-72-7	Analgesics	Surface Water	2014	123	ng/L	[1]
Salicylic acid	69-72-7	Analgesics	Surface Water	2013	128	ng/L	[1]
Salicylic acid	69-72-7	Analgesics	Surface Water	2015	128,2	ng/L	[16]
Salicylic acid	69-72-7	Analgesics	Surface Water	2014	129	ng/L	[1]
Salicylic acid	69-72-7	Analgesics	Surface Water	2014	130	ng/L	[1]
Salicylic acid	69-72-7	Analgesics	Surface Water	2013	137	ng/L	[1]
Salicylic acid	69-72-7	Analgesics	Surface Water	2014	139	ng/L	[1]
Salicylic acid	69-72-7	Analgesics	Surface Water	2013	165	ng/L	[1]
Salicylic acid	69-72-7	Analgesics	Surface Water	2013	165	ng/L	[1]
Salicylic acid	69-72-7	Analgesics	Surface Water	2013	172	ng/L	[1]
Salicylic acid	69-72-7	Analgesics	Surface Water	2014	190	ng/L	[1]
Salicylic acid	69-72-7	Analgesics	Surface Water	2013	198	ng/L	[1]
Salicylic acid	69-72-7	Analgesics	Surface Water	2013	209	ng/L	[1]
Salicylic acid	69-72-7	Analgesics	Surface Water	2013	209	ng/L	[1]
Salicylic acid	69-72-7	Analgesics	Surface Water	2013	214	ng/L	[1]
Salicylic acid	69-72-7	Analgesics	Surface Water	2014	217	ng/L	[1]
Salicylic acid	69-72-7	Analgesics	Surface Water	2013	232	ng/L	[1]
Salicylic acid	69-72-7	Analgesics	Surface Water	2014	294	ng/L	[1]
Salicylic acid	69-72-7	Analgesics	Surface Water	2019	54,6	ng/L	[1]
Salicylic acid	69-72-7	Analgesics	Surface Water	2019	74,9	ng/L	[1]
Salicylic acid	69-72-7	Analgesics	Surface Water	2019	75,4	ng/L	[1]
Salicylic acid	69-72-7	Analgesics	Surface Water	2019	85	ng/L	[1]
Salicylic acid	69-72-7	Analgesics	Surface Water	2018	106	ng/L	[1]
Salicylic acid	69-72-7	Analgesics	Surface Water	2018	118	ng/L	[1]
Salicylic acid	69-72-7	Analgesics	Surface Water	2019	125	ng/L	[1]
Salicylic acid	69-72-7	Analgesics	Surface Water	2018	180	ng/L	[1]
Salicylic acid	69-72-7	Analgesics	Surface Water	2018	205	ng/L	[1]
Salicylic acid	69-72-7	Analgesics	Surface Water	2018	348	ng/L	[1]
Sertraline	79617-96-2	Antidepressant	Surface Water	2018	8,74	ng/L	[1]
Sertraline	79617-96-2	Antidepressant	Surface Water	2019	9,34	ng/L	[1]
Sertraline	79617-96-2	Antidepressant	Surface Water	2019	12,5	ng/L	[1]

Sertraline	79617-96-2	Antidepressant	Surface Water	2019	21,4	ng/L	[1]
Sertraline	79617-96-2	Antidepressant	Surface Water	2017	5,4	ng/L	[8]
Sertraline	79617-96-2	Antidepressant	Surface Water	2014	23,3	ng/L	[9]
Simvastatin	79902-63-9	Statine	Surface Water	2009	42,9	ng/L	[10]
Sotalol	3930-20-9	beta blockers	Surface Water	2024	5,2	ng/L	[6]
Sotalol	3930-20-9	beta blockers	Surface Water	2024	8,1	ng/L	[6]
Sotalol	3930-20-9	beta blockers	Surface Water	2024	11,5	ng/L	[6]
Sulfadiazine	68-35-9	Antibiotic	Bottled water	2013	0,4	ng/L	[7]
Sulfadiazine	68-35-9	Antibiotic	Surface Water	2019	114	ng/L	[1]
Sulfadiazine	68-35-9	Antibiotic	Bottled water	2013	1	ng/L	[7]
Sulfadimethoxine	122-11-2	Antibiotics	Surface Water	2018	0,09	ng/L	[14]
Sulfadimethoxine	122-11-2	Antibiotics	Surface Water	2018	0,08	ng/L	[14]
Sulfadimethoxine	122-11-2	Antibiotics	Surface Water	2018	0,07	ng/L	[14]
Sulfadimethoxine	122-11-2	Antibiotics	Surface Water	2018	0,07	ng/L	[14]
Sulfadimethoxine	122-11-2	Antibiotics	Surface Water	2018	0,04	ng/L	[14]
Sulfadimethoxine	122-11-2	Antibiotics	Surface Water	2018	0,04	ng/L	[14]
Sulfadimethoxine	122-11-2	Antibiotics	Surface Water	2018	0,06	ng/L	[14]
Sulfadimethoxine	122-11-2	Antibiotics	Surface Water	2018	0,06	ng/L	[14]
Sulfadimethoxine	122-11-2	Antibiotics	Surface Water	2018	0,05	ng/L	[14]
Sulfadimethoxine	122-11-2	Antibiotics	Surface Water	2018	0,05	ng/L	[14]
Sulfadimethoxine	122-11-2	Antibiotics	Surface Water	2018	0,05	ng/L	[14]
Sulfadimethoxine	122-11-2	Antibiotics	Surface Water	2018	0,05	ng/L	[14]
Sulfadimethoxine	122-11-2	Antibiotics	Surface Water	2018	0,05	ng/L	[14]
Sulfadimethoxine	122-11-2	Antibiotics	Surface Water	2018	0,06	ng/L	[14]
Sulfadimethoxine	122-11-2	Antibiotics	Surface Water	2018	0,05	ng/L	[14]
Sulfadimethoxine	122-11-2	Antibiotics	Surface Water	2018	0,04	ng/L	[14]
Sulfadimethoxine	122-11-2	Antibiotics	Surface Water	2024	2,1	ng/L	[6]
Sulfadimethoxine	122-11-2	Antibiotics	Surface Water	2024	5,3	ng/L	[6]
Sulfadimethoxine	122-11-2	Antibiotics	Surface Water	2024	8,4	ng/L	[6]
Sulfamethazine	57-68-1	Antibiotics	Surface Water	2019	4,87	ng/L	[1]
Sulfamethazine	57-68-1	Antibiotics	Surface Water	2013	25,7	ng/L	[1]
Sulfamethazine	57-68-1	Antibiotics	Surface Water	2014	30	ng/L	[1]
Sulfamethazine	57-68-1	Antibiotics	Surface Water	2014	36,3	ng/L	[1]
Sulfamethazine	57-68-1	Antibiotics	Surface Water	2013	45,4	ng/L	[1]
Sulfamethazine	57-68-1	Antibiotics	Surface Water	2013	45,4	ng/L	[2]
Sulfamethazine	57-68-1	Antibiotics	Surface Water	2013	67,4	ng/L	[2]
Sulfamethazine	57-68-1	Antibiotics	Surface Water	2014	69,7	ng/L	[1]
Sulfamethazine	57-68-1	Antibiotics	Surface Water	2013	123	ng/L	[2]
Sulfamethoxazole	723-46-6	Antibiotics	Surface Water	2019	6,96	ng/L	[1]
Sulfamethoxazole	723-46-6	Antibiotics	Surface Water	2019	22,1	ng/L	[1]
Sulfamethoxazole	723-46-6	Antibiotics	Surface Water	2013	43	ng/L	[2]
Sulfamethoxazole	723-46-6	Antibiotics	Surface Water	2018	0,8	ng/L	[14]
Sulfamethoxazole	723-46-6	Antibiotics	Surface Water	2018	0,78	ng/L	[14]
Sulfamethoxazole	723-46-6	Antibiotics	Surface Water	2018	0,64	ng/L	[14]
Sulfamethoxazole	723-46-6	Antibiotics	Surface Water	2018	0,71	ng/L	[14]
Sulfamethoxazole	723-46-6	Antibiotics	Surface Water	2018	0,44	ng/L	[14]
Sulfamethoxazole	723-46-6	Antibiotics	Surface Water	2018	0,48	ng/L	[14]
Sulfamethoxazole	723-46-6	Antibiotics	Surface Water	2018	0,65	ng/L	[14]
Sulfamethoxazole	723-46-6	Antibiotics	Surface Water	2018	0,69	ng/L	[14]
Sulfamethoxazole	723-46-6	Antibiotics	Surface Water	2018	0,65	ng/L	[14]

Sulfamethoxazole	723-46-6	Antibiotics	Surface Water	2018	0,67	ng/L	[14]
Sulfamethoxazole	723-46-6	Antibiotics	Surface Water	2018	12,9	ng/L	[14]
Sulfamethoxazole	723-46-6	Antibiotics	Surface Water	2018	5,68	ng/L	[14]
Sulfamethoxazole	723-46-6	Antibiotics	Surface Water	2018	7,49	ng/L	[14]
Sulfamethoxazole	723-46-6	Antibiotics	Surface Water	2018	7,58	ng/L	[14]
Sulfamethoxazole	723-46-6	Antibiotics	Surface Water	2018	7,08	ng/L	[14]
Sulfamethoxazole	723-46-6	Antibiotics	Surface Water	2018	7,17	ng/L	[14]
Sulfamethoxazole	723-46-6	Antibiotics	Surface Water	2007	9,14	ng/L	[18]
Sulfamethoxazole	723-46-6	Antibiotics	Surface Water	2007	53,3	ng/L	[18]
Sulfamethoxazole	723-46-6	Antibiotics	Surface Water	2007	30,5	ng/L	[18]
Sulfapyridine	144-83-2	Antibiotic	Surface Water	2018	11,6	ng/L	[1]
Sulfapyridine	144-83-2	Antibiotic	Surface Water	2019	15,2	ng/L	[1]
Sulfapyridine	144-83-2	Antibiotic	Bottled water	2013	1	ng/L	[7]
Sulfapyridine	144-83-2	Antibiotic	Bottled water	2013	2	ng/L	[7]
Tetracycline	60-54-8	Antibiotic	Surface Water	2018	55,1	ng/L	[1]
Tiamulin	55297-95-5	Antibiotics	Surface Water	2018	0,07	ng/L	[14]
Tiamulin	55297-95-5	Antibiotics	Surface Water	2018	0,05	ng/L	[14]
Tiamulin	55297-95-5	Antibiotics	Surface Water	2018	0,1	ng/L	[14]
Tiamulin	55297-95-5	Antibiotics	Surface Water	2018	0,06	ng/L	[14]
Tiamulin	55297-95-5	Antibiotics	Surface Water	2018	0,02	ng/L	[14]
Tiamulin	55297-95-5	Antibiotics	Surface Water	2018	0,06	ng/L	[14]
Topiramate	97240-79-4	Anticonvulsant	Surface Water	2018	0,66	ng/L	[1]
Topiramate	97240-79-4	Anticonvulsant	Surface Water	2018	23,4	ng/L	[1]
Topiramate	97240-79-4	Anticonvulsant	Surface Water	2019	24,5	ng/L	[1]
Topiramate	97240-79-4	Anticonvulsant	Surface Water	2019	35,8	ng/L	[1]
Topiramate	97240-79-4	Anticonvulsant	Surface Water	2018	77,4	ng/L	[1]
Topiramate	97240-79-4	Anticonvulsant	Surface Water	2018	192	ng/L	[1]
Topiramate	97240-79-4	Anticonvulsant	Surface Water	2019	237	ng/L	[1]
Tramadol	27203-92-5	Opioid	Surface Water	2018	4444	ng/L	[12]
Tramadol	27203-92-5	Opioid	Surface Water	2024	8,7	ng/L	[6]
Tramadol	27203-92-5	Opioid	Surface Water	2024	61,8	ng/L	[6]
Tramadol	27203-92-5	Opioid	Surface Water	2024	1988	ng/L	[6]
Trazodone	19794-93-5	Antidepressant	Surface Water	2018	2,15	ng/L	[1]
Trazodone	19794-93-5	Antidepressant	Surface Water	2013	5,42	ng/L	[1]
Trazodone	19794-93-5	Antidepressant	Surface Water	2014	7,14	ng/L	[1]
Trazodone	19794-93-5	Antidepressant	Surface Water	2014	11,6	ng/L	[1]
Trazodone	19794-93-5	Antidepressant	Surface Water	2019	15,8	ng/L	[1]
Trazodone	19794-93-5	Antidepressant	Surface Water	2013	27,6	ng/L	[2]
Trazodone	19794-93-5	Antidepressant	Surface Water	2019	34	ng/L	[1]
Trazodone	19794-93-5	Antidepressant	Surface Water	2018	35,2	ng/L	[1]
Trazodone	19794-93-5	Antidepressant	Surface Water	2019	148	ng/L	[1]
Trimethoprim	738-70-5	Antibiotics	Surface Water	2019	80,6	ng/L	[1]
Trimethoprim	738-70-5	Antibiotics	Surface Water	2017	110	ng/L	[8]
Trimethoprim	738-70-5	Antibiotics	Surface Water	2018	38,1	ng/L	[8]
Trimethoprim	738-70-5	Antibiotics	Surface Water	2007	3,89	ng/L	[18]
Trimethoprim	738-70-5	Antibiotics	Surface Water	2007	15,7	ng/L	[18]
Trimethoprim	738-70-5	Antibiotics	Surface Water	2007	11,5	ng/L	[18]
Venlafaxine	93413-69-5	Antidepressant	Surface Water	2018	3,85	ng/L	[1]
Venlafaxine	93413-69-5	Antidepressant	Surface Water	2018	6,19	ng/L	[1]
Venlafaxine	93413-69-5	Antidepressant	Surface Water	2019	8,26	ng/L	[1]

Venlafaxine	93413-69-5	Antidepressant	Surface Water	2018	9,34	ng/L	[1]
Venlafaxine	93413-69-5	Antidepressant	Surface Water	2013	17,5	ng/L	[1]
Venlafaxine	93413-69-5	Antidepressant	Surface Water	2014	37,2	ng/L	[1]
Venlafaxine	93413-69-5	Antidepressant	Surface Water	2013	43	ng/L	[1]
Venlafaxine	93413-69-5	Antidepressant	Surface Water	2018	45,6	ng/L	[1]
Venlafaxine	93413-69-5	Antidepressant	Surface Water	2014	51,5	ng/L	[1]
Venlafaxine	93413-69-5	Antidepressant	Surface Water	2013	65	ng/L	[1]
Venlafaxine	93413-69-5	Antidepressant	Surface Water	2013	66,7	ng/L	[2]
Venlafaxine	93413-69-5	Antidepressant	Surface Water	2013	92,1	ng/L	[2]
Venlafaxine	93413-69-5	Antidepressant	Surface Water	2018	124	ng/L	[1]
Venlafaxine	93413-69-5	Antidepressant	Surface Water	2013	159	ng/L	[1]
Venlafaxine	93413-69-5	Antidepressant	Surface Water	2017	641	ng/L	[8]
Venlafaxine	93413-69-5	Antidepressant	Surface Water	2017	235	ng/L	[8]
Venlafaxine	93413-69-5	Antidepressant	Surface Water	2024	3,3	ng/L	[6]
Venlafaxine	93413-69-5	Antidepressant	Surface Water	2024	32,2	ng/L	[6]
Venlafaxine	93413-69-5	Antidepressant	Surface Water	2024	61,1	ng/L	[6]
Warfarin	81-81-2	Anticoagulant	Tap Water	2015	3,89	ng/L	[15]
Warfarin	81-81-2	Anticoagulant	Tap Water	2015	0,39	ng/L	[15]
Warfarin	81-81-2	Anticoagulant	Bottled water	2015	4,07	ng/L	[15]
Warfarin	81-81-2	Anticoagulant	Surface Water	2024	2,2	ng/L	[6]
Warfarin	81-81-2	Anticoagulant	Bottled water	2015	11,2	ng/L	[15]

1. Paíga P, Correia-Sá L, Correia M, Figueiredo S, Vieira J, Jorge S, et al. Temporal Analysis of Pharmaceuticals as Emerging Contaminants in Surface Water and Wastewater Samples: A Case Study. J Xenobiot. Multidisciplinary Digital Publishing Institute (MDPI); 2024;14(3):873–92. DOI: 10.3390/jox14030048

2. Paíga P, Santos LHMLM, Ramos S, Jorge S, Silva JG, Delerue-Matos C. Presence of pharmaceuticals in the Lis river (Portugal): Sources, fate and seasonal variation. Science of the Total Environment. Elsevier B.V.; 2016;573:164–77. DOI: 10.1016/j.scitotenv.2016.08.089

3. Lima DLD, Silva CP, Otero M, Esteves VI. Low cost methodology for estrogens monitoring in water samples using dispersive liquid-liquid microextraction and HPLC with fluorescence detection. Talanta. Elsevier B.V.; 2013;115:980–5. DOI: 10.1016/j.talanta.2013.07.007

4. Rocha MJ, Ribeiro M, Ribeiro C, Couto C, Cruzeiro C, Rocha E. Endocrine disruptors in the Leça River and nearby Porto Coast (NW Portugal): Presence of estrogenic compounds and hypoxic conditions. Toxicol Environ Chem. 2012;94(2):262–74. DOI: 10.1080/02772248.2011.644291

5. Rocha MJ, Cruzeiro C, Rocha E. Quantification of 17 endocrine disruptor compounds and their spatial and seasonal distribution in the Iberian Ave River and its coastline. Toxicol Environ Chem. 2013;95(3):386–99. DOI: 10.1080/02772248.2013.773002

6. Voznakova A, Antao-Geraldes AM, Canle M. Pharmaceuticals in the Douro basin: Occurrence, distribution, and ecological risk. J Environ Chem Eng. Elsevier Ltd; 2025;13(6). DOI: 10.1016/j.jece.2025.119181

7. Gaffney VJ, Almeida CMM, Rodrigues A, Ferreira E, Benoliel MJ, Cardoso VV. Occurrence of pharmaceuticals in a water supply system and related human health risk assessment. Water Res. Elsevier Ltd; 2015;72:199–208. DOI: 10.1016/j.watres.2014.10.027

8. Fernandes MJ, Paíga P, Silva A, Llaguno CP, Carvalho M, Vázquez FM, et al. Antibiotics and antidepressants occurrence in surface waters and sediments collected in the north of Portugal. Chemosphere. Elsevier Ltd; 2020;239. DOI: 10.1016/j.chemosphere.2019.124729

9. Pereira AMPT, Silva LJG, Laranjeiro CSM, Meisel LM, Lino CM, Pena A. Human pharmaceuticals in Portuguese rivers: The impact of water scarcity in the environmental risk. Science of the Total Environment. Elsevier B.V.; 2017;609:1182–91. DOI: 10.1016/j.scitotenv.2017.07.200

10. Gonçalves CMO, Sousa MAD, Alpendurada M de FPSP. Analysis of acidic, basic and neutral pharmaceuticals in river waters: Clean-up by 1°, 2° amino anion exchange and enrichment using an hydrophilic adsorbent. Int J Environ Anal Chem. 2013;93(1):1–22. DOI: 10.1080/03067319.2012.702272

11. Muñiz-Bustamante L, Caballero-Casero N, Rubio S. Drugs of abuse in tap water from eight European countries: Determination by use of supramolecular solvents and tentative evaluation of risks to human health. Environ Int. Elsevier Ltd; 2022;164. DOI: 10.1016/j.envint.2022.107281

12. Coelho MM, Lado Ribeiro AR, Sousa JCG, Ribeiro C, Fernandes C, Silva AMT, et al. Dual enantioselective LC–MS/MS method to analyse chiral drugs in surface water: Monitoring in Douro River estuary. J Pharm Biomed Anal. Elsevier B.V.; 2019;170:89–101. DOI: 10.1016/j.jpba.2019.03.032

13. Foureaux AFS, Reis EO, Lebron Y, Moreira V, Santos L v., Amaral MS, et al. Rejection of pharmaceutical compounds from surface water by nanofiltration and reverse osmosis. Sep Purif Technol. Elsevier B.V.; 2019;212:171–9. DOI: 10.1016/j.seppur.2018.11.018

14. Kötke D, Gandrass J, Bento CPM, Ferreira CSS, Ferreira AJD. Occurrence and environmental risk assessment of pharmaceuticals in the Mondego river (Portugal). Heliyon. Elsevier Ltd; 2024;10(15). DOI: 10.1016/j.heliyon.2024.e34825

15. Barbosa MO, Ribeiro AR, Pereira MFR, Silva AMT. Eco-friendly LC–MS/MS method for analysis of multi-class micropollutants in tap, fountain, and well water from northern Portugal. Anal Bioanal Chem. Springer Verlag; 2016;408(29):8355–67. DOI: 10.1007/s00216-016-9952-7

16. Paíga P, Santos LHMLM, Delerue-Matos C. Development of a multi-residue method for the determination of human and veterinary pharmaceuticals and some of their metabolites in aqueous environmental matrices by SPE-UHPLC–MS/MS. J Pharm Biomed Anal. Elsevier B.V.; 2017;135:75–86. DOI: 10.1016/j.jpba.2016.12.013

17.Calisto V, Bahlmann A, Schneider RJ, Esteves VI. Application of an ELISA to the quantification of carbamazepine in ground, surface and wastewaters and validation with LC-MS/MS. Chemosphere. Elsevier Ltd; 2011;84(11):1708–15. DOI: 10.1016/j.chemosphere.2011.04.072

18. Madureira TV, Barreiro JC, Rocha MJ, Rocha E, Cass QB, Tiritan ME. Spatiotemporal distribution of pharmaceuticals in the Douro River estuary (Portugal). Science of the Total Environment. 2010;408(22):5513–20. DOI: 10.1016/j.scitotenv.2010.07.069

19. Pena A, Chmielova D, Lino CM, Solich P. Determination of fluoroquinolone antibiotics in surface waters from Mondego River by high performance liquid chromatography using a monolithic column. J Sep Sci. 2007;30(17):2924–8. DOI: 10.1002/jssc.200700363

20. Sousa MA, Gonçalves C, Cunha E, Hajšlová J, Alpendurada MF. Cleanup strategies and advantages in the determination of several therapeutic classes of pharmaceuticals in wastewater samples by SPE-LC-MS/MS. Anal Bioanal Chem. 2011;399(2):807–22. DOI: 10.1007/s00216-010-4297-0

21. Paíga P, Santos LHMLM, Amorim CG, Araújo AN, Montenegro MCBSM, Pena A, et al. Pilot monitoring study of ibuprofen in surface waters of north of Portugal. Environmental Science and Pollution Research. Springer Verlag; 2013;20(4):2410–20. DOI: 10.1007/s11356-012-1128-1

22. Santos LHMLM, Paíga P, Araújo AN, Pena A, Delerue-Matos C, Montenegro MCBSM. Development of a simple analytical method for the simultaneous determination of paracetamol, paracetamol-glucuronide and p-aminophenol in river water. J Chromatogr B Analyt Technol Biomed Life Sci. 2013;930:75–81. DOI: 10.1016/j.jchromb.2013.04.032

Table S2. Database of acceptable daily intake values for each pharmaceutical residue evaluated in this study.

Pharmaceutical compound	ADI (µg/kg/d)	Reference
10,11-Epoxycarbamazepine	2.9	[1]
17-alpha-Ethinylestradiol	0.0001	[2]
17-beta-Estradiol	0.0003	[2]
Acetaminophen	3.1	[3]
Amoxicillin	0.5	[2]
Atenolol	0.8	[2]
Atorvastatin	0.17	[2]
Azithromycin	3.0	[2]
Benzoylecgonine	-	[4]
Betamethasone	0.008	[2]
Bezafibrate	10	[2]
Bisoprolol	1.5	[5]
Bupropion	57.1	[6]
Carbamazepine	0.3	[7]
Carboxyibuprofen	13.3	[2]
Cetirizine	0.08	[8]
Chlorfenvinphos	27.5	[9]
Ciprofloxacin	0.48	[3]
Citalopram	0.0008	[10]
Citalopram propionic acid	0.0008	[10]
Clarithromycin	8.3	[2]
Clofibric	10	[2]
Cocaine	-	[4]
Codeine	2	[11]
Diazepam	1	[1]
Diclofenac	0.5	[2]
Diltiazem	2.0	[2]
Enrofloxacin	6.2	[2]
Erythromycin	1.2	[3]
Estrone	0.0003	[2]
Fenofibrate acid	2000	[12]
Fluconazole	0.8	[2]
Fluoxetine	2.9	[11]
Furosemide	0.14	[13]
Gemfibrozil	20	[2]
Hydrochlorothiazide	0.21	[2]
Hydroxyibuprofen	13.3	[2]
Ibuprofen	13.3	[2]
Iohexol	125000	[1]
Iomeprol	1900	[14]
Iopamidol	118600	[1]
Iopromide	710	[14]
Isoniazid	4.3	[13]
Ketoprofen	1	[2]
Lincomycin	25	[11]
Lorazepam	0.00071	[13]

Metformin	8.3	[2]
Naproxen	7.3	[2]
Nimesulide	3.3	[2]
Ofloxacin	5.7	[15]
Omeprazole	0.17	[2]
p-Aminophenol	3.1	[3]
Paracetamol-glucuronide	3.1	[3]
Paroxetine	2.9	[11]
Phenylbutazone	200	[16]
Prednisone	0.042	[2]
Primidone	0.7	[1]
Propranolol	0.5	[2]
Ramipril	0.0018	[13]
Roxithromycin	2.1	[13]
Salicylic acid	8.3	[17]
Sertraline	0.036	[13]
Simvastatin	0.08	[2]
Sotalol	2.3	[13]
Sulfadiazine	9.5	[3]
Sulfadimethoxine	6	[18]
Sulfamethazine	9.5	[3]
Sulfamethoxazole	3.8	[3]
Sulfapyridine	4.8	[3]
Tetracycline	30	[2]
Tiamulin	250	[19]
Topiramate	100	[20]
Tramadol	7.1	[1]
Trazodone	0.029	[13]
Trimethoprim	1.7	[2]
Venlafaxine	5.4	[1]
Warfarin	0.16	[11]

1. Jurado, A.; Labad, F.; Scheiber, L.; Criollo, R.; Nikolenko, O.; Pérez, S.; Ginebreda, A. Occurrence of Pharmaceuticals and Risk Assessment in Urban Groundwater. Adv. Geosci. 2022, 59, 1–7, doi:10.5194/adgeo-59-1-2022.

2. de Aquino, S.F.; Brandt, E.M.F.; Bottrel, S.E.C.; Gomes, F.B.R.; Silva, S. de Q. Occurrence of Pharmaceuticals and Endocrine Disrupting Compounds in Brazilian Water and the Risks They May Represent to Human Health. IJERPH 2021, 18, 11765, doi:10.3390/ijerph182211765.

3. Semerjian, L.; Shanableh, A.; Semreen, M.H.; Samarai, M. Human Health Risk Assessment of Pharmaceuticals in Treated Wastewater Reused for Non-Potable Applications in Sharjah, United Arab Emirates. Environment International 2018, 121, 325–331, doi:10.1016/j.envint.2018.08.048.

4. National Institute on Drug Abuse (NIDA). Cocaine Research Report: Is There a Safe Dose of Cocaine? Available at: https://nida.nih.gov/publications/research-reports/cocaine (Acess in May 5, 2026).

5. Simon, S.; Schlingemann, J.; Johnson, G.; Brenneis, C.; Guessregen, B.; Kostal, J.; Dieckhoff, J. Deriving Safe Limits for N-Nitroso-Bisoprolol by Error-Corrected next-Generation Sequencing (ecNGS) and Benchmark Dose (BMD) Analysis, Integrated with QM Modeling and CYP-Docking Analysis. Arch Toxicol 2025, 99, 3935–3962, doi:10.1007/s00204-025-04103-2.

6. Cunningham, V.L.; Binks, S.P.; Olson, M.J. Human Health Risk Assessment from the Presence of Human Pharmaceuticals in the Aquatic Environment. Regulatory Toxicology and Pharmacology 2009, 53, 39–45, doi:10.1016/j.yrtph.2008.10.006.

7. Bruce, G.M.; Pleus, R.C.; Snyder, S.A. Toxicological Relevance of Pharmaceuticals in Drinking Water. Environ. Sci. Technol. 2010, 44, 5619–5626, doi:10.1021/es1004895.

8. Ogolla Wanjeri, V.W.; Okuku, E.; Ngila, J.C.; Waiyaki, E.; Nyingi, J.K.; Ndungu, P.G. Occurrence and Distribution of Selected Pharmaceuticals in Fresh Fish along the Kenyan Coast and Assessment of Potential Human Health Risks. Environ. Sci.: Adv. 2025, 4, 938–951, doi:10.1039/d4va00392f.

9. Chun, O.K.; Kang, H.G. Estimation of Risks of Pesticide Exposure, by Food Intake, to Koreans. Food and Chemical Toxicology 2003, 41, 1063–1076, doi:10.1016/s0278-6915(03)00044-9.

10. Silva, L.J.G.; Pereira, A.M.P.T.; Rodrigues, H.; Meisel, L.M.; Lino, C.M.; Pena, A. SSRIs Antidepressants in Marine Mussels from Atlantic Coastal Areas and Human Risk Assessment. Science of The Total Environment 2017, 603–604, 118–125, doi:10.1016/j.scitotenv.2017.06.076.

11. Schwab, B.W.; Hayes, E.P.; Fiori, J.M.; Mastrocco, F.J.; Roden, N.M.; Cragin, D.; Meyerhoff, R.D.; D'Aco, V.J.; Anderson, P.D. Human Pharmaceuticals in US Surface Waters: A Human Health Risk Assessment. Regulatory Toxicology and Pharmacology 2005, 42, 296–312, doi:10.1016/j.yrtph.2005.05.005.

12. Bercu, J.P.; Jolly, R.A.; Flagella, K.M.; Baker, T.K.; Romero, P.; Stevens, J.L. Toxicogenomics and Cancer Risk Assessment: A Framework for Key Event Analysis and Dose–Response Assessment for Nongenotoxic Carcinogens. Regulatory Toxicology and Pharmacology 2010, 58, 369–381, doi:10.1016/j.yrtph.2010.08.002.

13. Khan, U.; Nicell, J. Human Health Relevance of Pharmaceutically Active Compounds in Drinking Water. AAPS J 2015, 17, 558–585, doi:10.1208/s12248-015-9729-5.

14. Schriks, M.; Heringa, M.B.; van der Kooi, M.M.E.; de Voogt, P.; van Wezel, A.P. Toxicological Relevance of Emerging Contaminants for Drinking Water Quality. Water Research 2010, 44, 461–476, doi:10.1016/j.watres.2009.08.023.

15. Prosser, R.S.; Sibley, P.K. Human Health Risk Assessment of Pharmaceuticals and Personal Care Products in Plant Tissue Due to Biosolids and Manure Amendments, and Wastewater Irrigation. Environment International 2015, 75, 223–233, doi:10.1016/j.envint.2014.11.020.

16. Infinity Pharma. Fenilbutazona. Available at: https://www.infinitypharma.com.br/wp-content/uploads/2023/06/Fenilbutazona.pdf (Acess in May 5, 2026).

17. Kumar, A.; Chang, B.; Xagoraraki, I. Human Health Risk Assessment of Pharmaceuticals in Water: Issues and Challenges Ahead. IJERPH 2010, 7, 3929–3953, doi:10.3390/ijerph7113929.

18. Zuo, X.; Ai-yun, H. The Residues and Risk Assessment of Sulfonamides in Animal Products. Journal of Food Quality 2021, 2021, 1–6, doi:10.1155/2021/5597755.

19. DGAV – Direção-Geral de Alimentação e Veterinária. CALIERMUTIN 125. Available at: https://medvet.dgav.pt/medvet_dgav/static/RCM/CALIERMUTIN_125.pdf (Acess in May 5, 2026).

20. Aché. Bula do Paciente – Topiramato. Available at: https://www.ache.com.br/wp-content/uploads/application/pdf/bula-paciente-topiramato.pdf (Acess in May 5, 2026).
